# Function-adaptive clustered nanoparticles reverse *Streptococcus mutans* dental biofilm and maintain microbiota balance

**DOI:** 10.1038/s42003-021-02372-y

**Published:** 2021-07-15

**Authors:** Fatemeh Ostadhossein, Parikshit Moitra, Esra Altun, Debapriya Dutta, Dinabandhu Sar, Indu Tripathi, Shih-Hsuan Hsiao, Valeriya Kravchuk, Shuming Nie, Dipanjan Pan

**Affiliations:** 1 Departments of Bioengineering, Beckman Institute, University of Illinois at Urbana-Champaign, Mills Breast Cancer Institute, and Carle Foundation Hospital, Urbana, IL USA; 2 Department of Pediatrics, Center for Blood Oxygen Transport and Hemostasis, Health Sciences Facility III, University of Maryland Baltimore School of Medicine, Baltimore, MD USA; 3 Veterinary Diagnostic Laboratory, University of Illinois at Urbana-Champaign, Urbana, IL USA; 4 Departments of Bioengineering, Carle Illinois College of Medicine, Beckman Institute, Department of Chemistry, Department of Materials Science and Engineering, University of Illinois at Urbana-Champaign, Urbana, IL USA; 5 Department of Diagnostic Radiology and Nuclear Medicine, Health Sciences Facility III, University of Maryland Baltimore, Baltimore, MD USA; 6 Department of Chemical, Biochemical and Environmental Engineering, University of Maryland Baltimore County, Interdisciplinary Health Sciences Facility, Baltimore, MD USA

**Keywords:** Drug delivery, Nanoparticles

## Abstract

Dental plaques are biofilms that cause dental caries by demineralization with acidogenic bacteria. These bacteria reside inside a protective sheath which makes any curative treatment challenging. We propose an antibiotic-free strategy to disrupt the biofilm by engineered clustered carbon dot nanoparticles that function in the acidic environment of the biofilms. In vitro and ex vivo studies on the mature biofilms of *Streptococcus mutans* revealed >90% biofilm inhibition associated with the contact-mediated interaction of nanoparticles with the bacterial membrane, excessive reactive oxygen species generation, and DNA fragmentation. An in vivo examination showed that these nanoparticles could effectively suppress the growth of *S. mutans*. Importantly, 16S rRNA analysis of the dental microbiota showed that the diversity and richness of bacterial species did not substantially change with nanoparticle treatment. Overall, this study presents a safe and effective approach to decrease the dental biofilm formation without disrupting the ecological balance of the oral cavity.

## Abstract (web summary)

Ostadhossein et al. propose an antibiotic-free strategy to disrupt the biofilm by utilizing nanoparticles designed to function in the acidic environment of the biofilms. Phophonium ions functionalised carbon dot nanoclusters in a pH-sensitive polymer shell display dental biofilm inhibition without disrupting the ecological balance of the oral cavity.

## Introduction

As a unique niche for microbiota, the oral cavity harbors a wide array of bacteria, yeasts, protozoa, and viruses. A perturbation in this community can result in a loss of mutualistic/symbiotic balance, leading to diseases including caries^1–4^. The aggregates of these microorganisms can deposit on the hard surfaces of teeth in an orderly manner, forming a protective sheath referred to as extracellular polymeric substance (EPS) and resulting in oral biofilms or dental plaques^5,6^. Inside the biofilm, the fermentation of dietary carbohydrates, in particular sugars, causes the production of organic acids with a pH ~4.5. These acids can demineralize the tooth enamel, resulting in caries^7,8^. Dental caries is the most prevalent chronic childhood condition showing a precipitous increase in occurrences, affecting 2.4 billion people and accounting for ~$544 billion in global expenditures in 2015 (ref. ^9^). There is mounting evidence that dental biofilms can also lead to other diseases, such as periodontitis and systemic ailments, such as respiratory and brain abscesses, diabetes mellitus, rheumatoid arthritis, and infective endocarditis^10–12^. The most common carious pathogen involved in dental biofilms is *Streptococcus mutans*, a gram-positive bacterium that is both acidogenic (i.e., produces acids) and aciduric (tolerates acids) and actively produces EPS^8,13–16^. Hence, this model bacterium has been used by several research groups in identifying the therapeutic options for the dental biofilm and to model the disease in vitro and in vivo due to its dominating nature^15–19^.

Unfortunately, effective treatments for dental caries are difficult to develop due to the multifactorial nature of the disease. Preventative strategies are currently being pursued in clinical settings, and little success in sustained EPS eradication has been achieved^1,20^. Examples of these strategies include mechanical debridement of the biofilm (e.g., brushing and irrigation), drug-eluting devices, and chemoprophylactic agents, such as fluoride^10,21–23^. A “gold standard” antiseptic agent in this regard is the cationic phenyl-bis biguanide chlorhexidine (CHX)^24,25^. This small molecule antibiotic interacts with negatively charged molecules (sulfates and phosphates) on the bacterial surface and disrupts membrane integrity, resulting in bacterial cell death^26^. However, despite its widespread use, the antiplaque and anticaries properties of CHX are highly disputed^27,28^. In addition, the long-term use of CHX leads to undesirable side effects, such as tartar formation, teeth staining, allergic reaction, and dysgeusia^27,28^. The widespread use of antibiotics also increases the likelihood of bacterial resistance development.

Nanoparticle (NP)-based approaches can be a much-desired solution to the biofilm issue as they offer several advantages, such as multifunctionality, controlled release of the drug, high loading efficiency, selectivity, and trackability^29–38^. Activatable NPs can be designed to work only in the presence of a trigger to minimize the off-target side effects^39–42^. This differs from conventional antibiotics, which indiscriminately target a wide range of bacterial populations, many of which are commensal and are crucial in fighting off pathogenic infections^43^. As a major group of nanobiotics, experimentation with metallic NPs has raised concerns, regarding their accumulation and non-degradation^44,45^. Many studies have shown that unlike mature biofilms, bacterial populations in the planktonic stage could be easily cleared by NP-based approaches^44,46,47^. Therefore, early and potent treatments for bacterial biofilms are desirable. However, the early diagnosis of bacterial infection is currently difficult, and most clinical bacterial infections are in fact mature biofilms, which are usually difficult to eradicate with antimicrobial treatments. Recently, we have shown that a high-affinity, pathogen-selective peptide can be employed to molecularly target the cariogenic pathogen *S. mutans* and be detected using X-ray imaging^48^. Thus, developing nano-enabled strategies for the treatment of biofilm infections is of great significance^49–51^.

Herein, we present for the first time a “particle-in-particle” approach for targeting the characteristic pH of EPS to localize and leverage inherently therapeutic NPs to kill *S. mutans*, as the most caries inducing bacteria^52^. More specifically, a large “nanoreservoir” containing  clusters of phosphonium-containing smaller therapeutic NPs  were wrapped in a smart, pH-responsive layer of an amphiphilic polymer, i.e., poly(styrene)-*b*-poly(*N*,*N*-dimethylaminoethyl methacrylate) (PS-*b*-PDMA) to facilitate the contact-mediated bacteria killing in the biofilm pH. The metabolic state of the dental biofilm in the low pH can initiate the pathogenicity, while targeting this factor can maintain the original ecological balance, as well as suppressing the harmful pathogens^53^. Phosphonium ions were adopted to elicit the NPs with antibacterial properties and a more pronounced inhibitory effect relative to their ammonium counterparts, as has been reported before^52^. The particle-in-particle approaches have been elegantly demonstrated in the past for the drug delivery purposes especially in the cancer treatment^42,54–56^. The delivery of small therapeutic NPs without using a secondary antibiotic via facile chemistry makes our approach unique and bars the need of multistep polymer synthesis.

Carbon dots (CDots) are an emerging class of carbon-based NPs that have attracted substantial research attention in recent years due to their ease of fabrication, tunable luminescent properties, and abundance of functional groups^57–65^. These properties combined with their degradability could offer a unique platform for combating bacteria^65,66^. Based on this premise, in this study, we demonstrate that dual action CDots could eliminate biofilm EPS and decrease *S. mutans* viability as an accurate representative biofilm-causing bacteria in an in vitro model of human teeth. The mechanism of action of the composite NPs can be classified as reactive oxygen species (ROS) generation and fragmentation of genomic DNA at the subcellular level. We further show that in vivo, these NPs markedly reduce the population of *S. mutans* without changing the diversity and richness of the bacterial microbiota, while preserving the oral ecological balance.

## Results and discussion

### Engineering of nanoparticles via assembly and physicochemical characterization

**Fig. 1:**
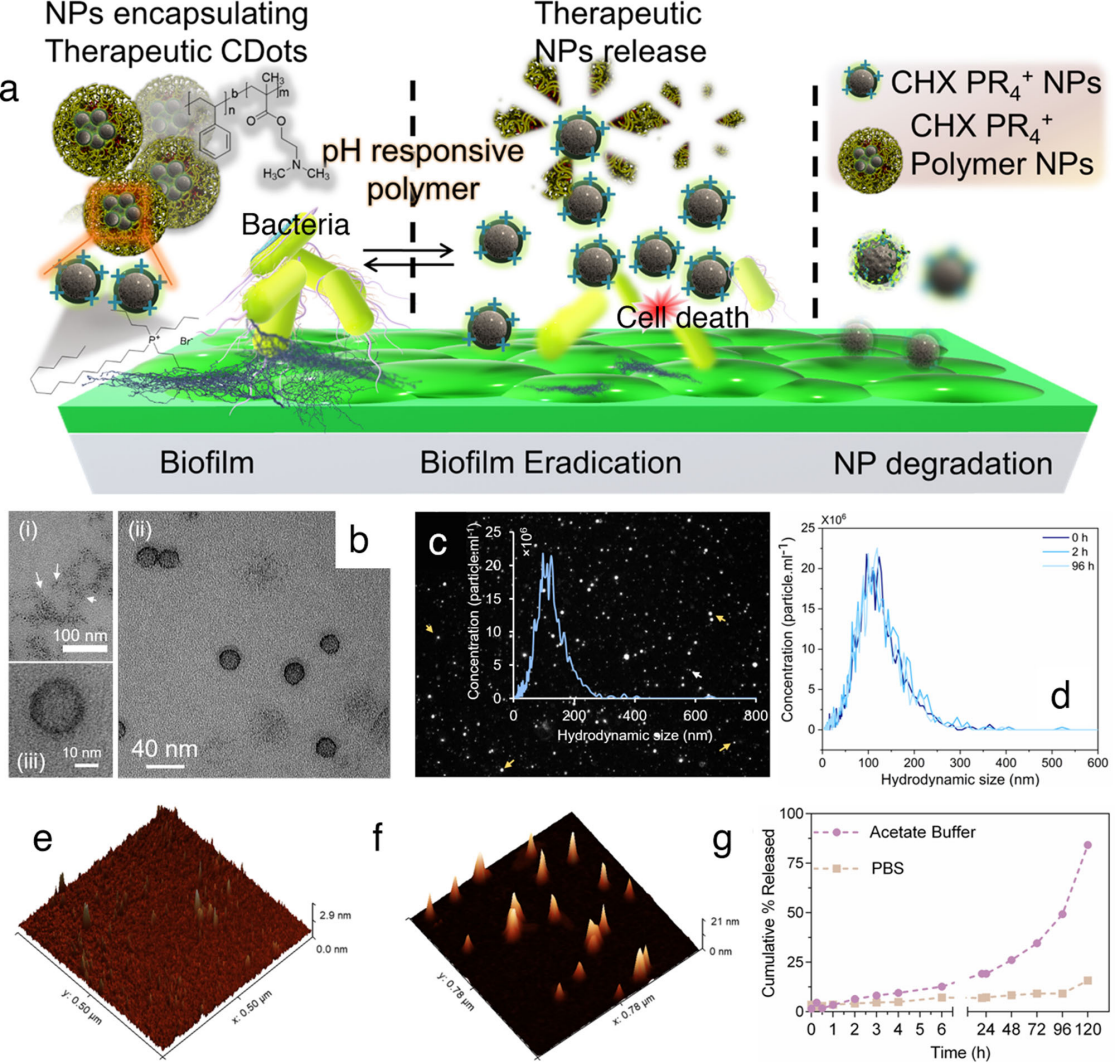
Design and characterization of nanoparticles. **a** Schematic representation of the action of NPs as antibiofilm agents. The inherently antibiofilm CDots functionalized with phosphonium ions were encapsulated in a pH-responsive diblock copolymer. At low pH, the shell will open to release the therapeutic load leading to the eradication of the dental biofilm, the zoomed region represents the wrapping of smaller CDots in the diblock copolymer, the NP degradation is represented as faded particles; **b** transmission electron microscopic image of (i) bare CHX PR_4_^+^ NPs, (ii) and (iii) polymer-wrapped CDots (CHX PR_4_^+^ polymer NPs); **c** Hydrodynamic distribution of CHX PR_4_^+^ polymer NPs measured by ZetaView, arrows point to NPs; **d** hydrodynamic size stability over time; AFM height variation **e** before and **f** after wrapping the CHX PR_4_^+^ NPs in the polymer; **g** pH-dependent release profile.

We adopted CHX as the CDot preparation source to minimize the variability between the control and our resultant NPs. As will be shown in the comprehensive experiments, we confirmed that CHX will not persist during the synthesis step, and hence makes this approach an antibiotic-free treatment relying on the inherent electrostatic interaction and downstream effects to eradicate the biofilm of *S. mutans*. This choice would also partially address the antibiotic resistance issue caused by the conventional commercially available antibiotics (Fig. 1a).

The CDots were synthesized using a one-pot hydrothermal method in an autoclave under high pressure (~2 MPa) at 180 °C. Briefly, CHX or CHX admixed with 30 wt% of tributylhexadecylphosphonium bromide (PR_4_^+^) was dissolved in methanol and exposed to heat and pressure for 24 h to afford the CHX NPs and CHX PR_4_^+^ NPs, respectively. To wrap the CDots (i.e., CHX NPs and/or CHX PR_4_^+^ NPs) in the pH-responsive polymer, a thin-film formation and hydration method was adopted^67^. Equal amounts of solid CDots from the above batches and the block copolymer (PS-*b*-PDMA) were dispersed in chloroform (CHCl_3_), vortexed vigorously, and sonicated for 5 min. Subsequently, a gentle stream of nitrogen gas was applied to remove the organic solvent, yielding a monolayer of CDots and PS_64_-*b*-PDMA_53_. After drying overnight to remove the residual organic solvent, 8 ml of water was added to adjust the concentration of CDots to 12.5 mg ml^−1^. The CHX NPs and CHX PR_4_^+^ NPs initially showed poor water dispersibility; however, the solubility was improved upon polymer wrapping. It is hypothesized that during the assembly process, the hydrophobic ethenylbenzene moieties in polystyrene would interact via π–π stacking with the hydrophobic CDots from CHX (due to the hydrophobicity of the precursor), while the PDMA, which has an abundance of hydrophilic tertiary amines^68^, would surface toward water and form micellar assembly entrapping multiple CDots.

**Fig. 2:**
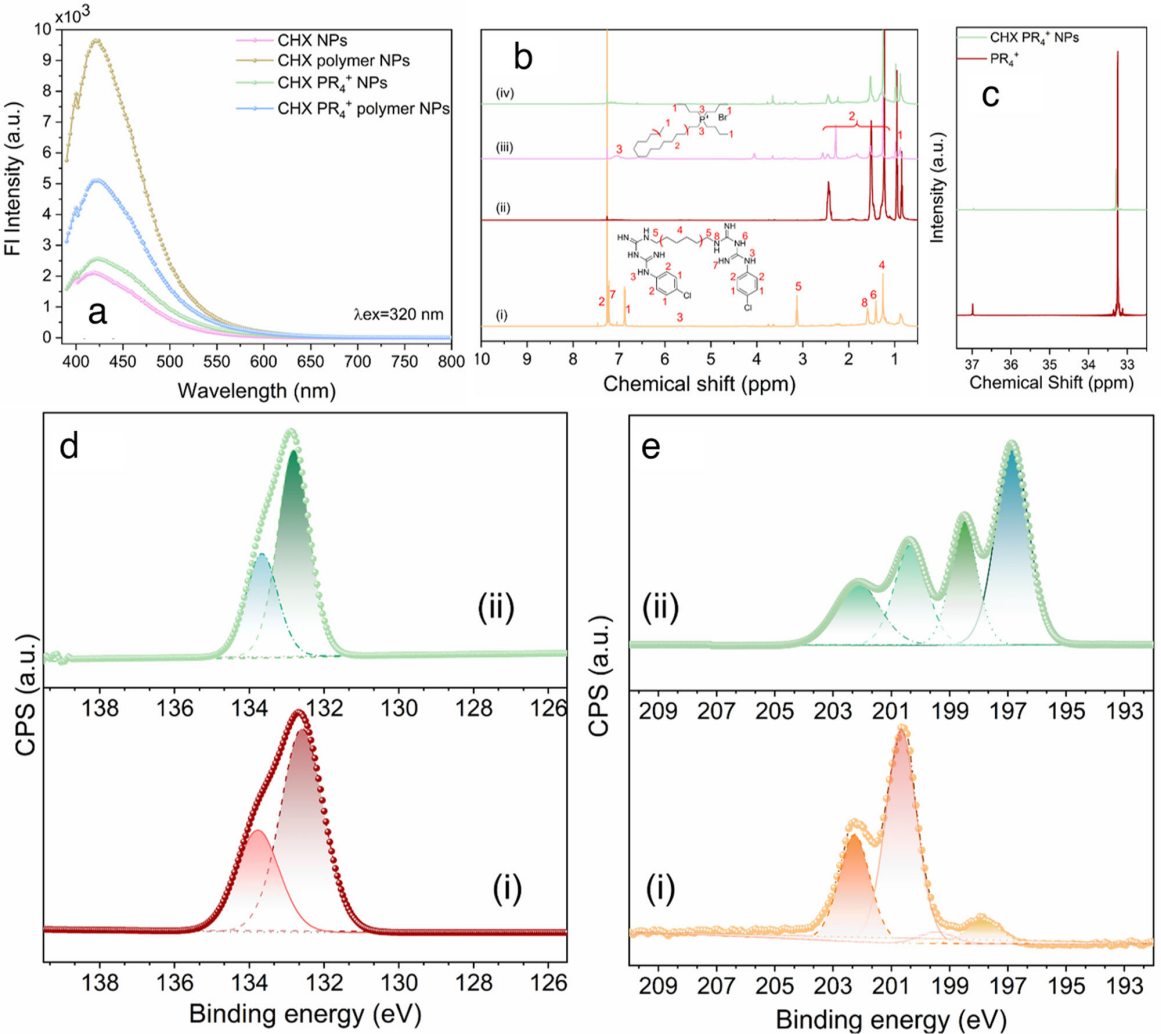
Characterization of chemical properties of NPs. **a** Fluorescence emission spectra of the NPs with and without polymer wrapping with ${\lambda }_{{\mathrm{excitation}}}=320\,{\mathrm{nm}}$. The signal from the CDots is preserved and enhanced when wrapped in the polymer shell; **b** ^1^H NMR spectra of (i) CHX, (ii) CHX NPs, (iii) PR_4_^+^ compound, and (iv) CHX PR_4_^+^ NPs and the corresponding assignments; **c** ^31^P NMR spectra comparing the phosphonium compound and CHX PR_4_^+^ NPs; **d** XPS spectra in the P-binding region of (i) phosphonium compound (PR_4_^+^) and (ii) CHX PR_4_^+^ NPs, which showed similar peaks; **e** XPS spectra in the Cl-binding region for (i) chlorhexidine (CHX) and (ii) CHX PR_4_^+^ NPs, which showed noticeable changes in the chemical binding.

The formation of NPs was initially confirmed by transmission electron microscopy (TEM), as shown in Fig. 1b). The CHX PR_4_^+^ NPs (Fig. 1b-i) and CHX PR_4_^+^ polymer NPs (Fig. 1b-ii, iii) have average anhydrous diameters of 2.6 ± 0.6 and 26 ± 4 nm, respectively, and are spherical. The CHX PR_4_^+^ polymer NPs are well-dispersed as can be concluded from TEM images, which shows anhydrous dried soft particles, while the original CHX PR_4_^+^ NPs generated poorer dispersion due to hydrophobic nature of these NPs. Indeed, to obtain proper images of the latter, we had to disperse them in organic solvents. The lower contrast of CDots can be attributed to their carbonaceous structure, which resembles the TEM grid and its low electron density. The NPs were reproducibly synthesized for four different batches at various instances and we got consistent results in all these batches. The hydrodynamic size distribution curve is indicated in Fig. 1c, which is based on the the analysis of a single particle rather than conventional autocorrelation calculations to determine the average particle size. The hydrodynamic size distribution was not affected overtime as shown in Fig. 1d. The difference in hydrodynamic size and anhydrous diameter is due to the loss of hydration layer when preparing TEM grids. The atomic force microscopy (AFM) results (Fig. 1e, f) demonstrated a considerable height increase after wrapping the individual CDots in the polymeric shell, indicating the entrapment of the smaller CDots. Moreover, the results of fluorescence spectroscopy (Fig. 2a) demonstrated the maintained and enhanced fluorescence signal of the CDots after wrapping in the diblock copolymer, which confirms the success of the entrapment. We also calculated nominally the average number of the CHX PR_4_^+^ NPs in the CHX PR_4_^+^ polymer NPs. The concentration of the CHX PR_4_^+^ polymer NP was measured to be 5.7 × 10^10^ using ZetaView system data for 2.5 mg ml^−1^ NPs. This result combined with the mass spectroscopy (MS) data (vida infra) yielded 7.4 × 10^10^ CHX PR_4_^+^ NPs per CHX PR_4_^+^ polymer NPs, as shown in “Methods” section.

The electrophoretic *ζ*-potential values for the CHX polymer NPs and CHX PR_4_^+^ polymer NPs were measured as −13 ± 3 and +35 ± 4 mV, respectively (Fig. S1). The magnitudes of these values were smaller than those of the corresponding CDots without the polymer, which is presumably due to the shielding effect of the polystyrene (i.e., the negative charge from polystyrene makes CHX polymer NPs more negative and reduces the positive charge of CHX PR_4_^+^ NPs). On the other hand, the absolute value for CHX PR_4_^+^ polymer NPs was greater than that of CHX polymer NPs, which resulted in a more colloidally stable system. The overall positive *ζ*-potential of CHX PR_4_^+^ NPs and CHX PR_4_^+^ polymer NPs confirm the successful incorporation of phosphonium functionalities in the CHX PR_4_^+^ NPs.

We exploited the pH responsiveness of the diblock copolymer for the controlled release of therapeutic CDot NPs. The pH of the biofilm is lower at the interface of the enamel and the bacterial colonies. Accordingly, the acidic regions were found only in the interior of microcolonies. The presence of highly acidic pH values within biofilms despite of the neutral-pH environment found in the oral cavity serves as a major virulence attribute for development of dental caries^69–71^. As shown in Fig. 1g, kinetics studies demonstrated that the relase rate is faster under acidic conditions (acetate buffer) commensurate with pH of the pathogenic biofilms^72^. This property could be attributed to the PDMA block in the diblock copolymer, which becomes fully protonated with extended chains, enabling the release of the cargo as has been shown in prior works along with the respective mechanism^73^. Moreover, wrapping of CHX PR_4_^+^ NPs in polymer reduces the offsite toxicity as a result of effective activity in the biofilm pH, and may potentially enhance the interaction with the bacterial membrane due to the hydrophobic core. It has been established that the presence of phenyl group in the macromolecule structures can improve the penetration in the bacterial membrane^74^. We believe that the internalization of smaller therapeutic NPs is via passive diffusion at the interface of the biofilm and the enamel. Therefore, NPs with this formulation were adopted for the antibiofilm experiments to achieve the desired outcome.

To elucidate the chemical structure of these NPs and determine whether the original source structure is preserved after the harsh carbonization step, we used several other characterization methods, namely, MS, nuclear magnetic resonance (NMR) spectroscopy, and X-ray photoelectron spectroscopy (XPS). Interestingly, the mass spectrometric results (Fig. S6) revealed that the major peak of CHX in drug form (*m*/*z* = 505.3) was drastically suppressed in the derived CHX NPs. In addition, the ^1^H NMR spectra indicated the lack of features from CHX after carbonization (Fig. 2b and Supplementary Data 1). ^31^P NMR analysis (Fig. 2c) revealed the presence of phosphorous moieties after NP formation. Finally, XPS was carried out to determine the chemical bonds formed during carbonization. For the CHX and CHX NPs, C, O, N, and Cl were detected (Figs. S7 and 8), whereas for PR_4_^+^ and CHX PR_4_^+^ NPs, C, N, O, Cl, P, and Br were detected (Figs. S9 and 10).

The decomposition of the phosphorus P2p core binding energy revealed a doublet peak similar to that of the phosphonium-containing precursor and CHX PR_4_^+^ NPs at 132.3 and 133.1 eV attributed to P 2p1/2 and P 2p3/2 in ionic phosphorous (Fig. 2d). For chlorine, the peak shapes and positions of the precursor were dramatically different from those of the NPs. The peaks for the drug, CHX, were detected at 200.6, 202.2, 197.8, and 199.4 eV, and these peaks shifted to 200.3, 202.15, 198.5, and 196.8 eV for CHX PR_4_^+^ NPs (Fig. 2e). The results collectively indicate that the properties of CHX changed, while the phosphonium content was maintained. Further characterizations on the surface functional groups are provided in the Supplementary Information (Figs. S2–10).

### Polymer-wrapped therapeutic nanoparticles kill *S. mutans* in vitro

**Fig. 3:**
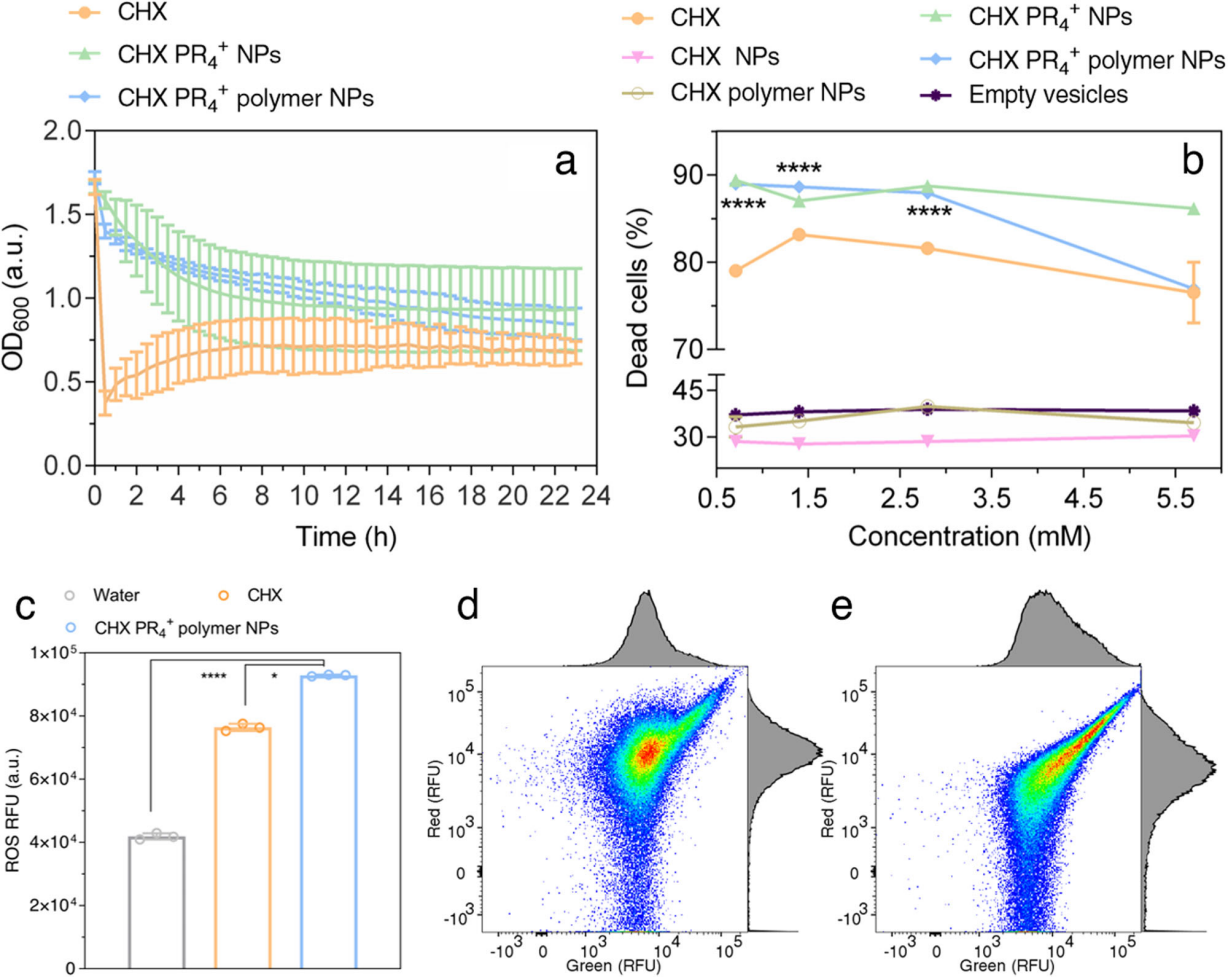
The efficacy of NPs against the planktonic form of *S. mutans*. **a** Turbidity time-kill assay for CHX, CHX PR_4_^+^ NPs, and CHX PR_4_^+^ polymer NPs showing a decreasing trend (*n* = 3); **b** the quantitative live–dead fluorescence assay for various formulations, *n* = 3, two-way ANOVA vs. CHX, *p* value < 0.0001 is indicated with ****; **c** ROS assay comparing the fluorescence intensities after the treatment with various formulations, ordinary one-way ANOVA, *n* = 3, *p* values < 0.1 and 0.001 are indicated by * and ****, respectively ; Error bar represents mean ± SD; **d**, **e** membrane potential measurements are determined by the ratio of red: green fluorescence in flow cytometry. The ratiometric parameters were 467 for **d** control and 204 for **e** treatment with CHX PR_4_^+^ polymer NPs.

We first examined the bactericidal properties of the NPs using a turbidity assay, which is typically utilized to rigorously characterize the dynamics of antibacterial properties^75,76^. *S. mutans* was exposed to various NP formulations, and the OD_600_ was recorded every 30 min. As shown in Fig. 3a and Supplementary Data 1, there is a constant decrease in bacterial viability upon treatment with CHX PR_4_^+^ NPs and CHX PR_4_^+^ polymer NPs, whereas for CHX NPs and CHX polymer NPs (Fig. S12), an exponential bacterial growth was observed despite an initial inhibition. The initial high value at *t* = 0 can be attributed to scattering effect. Therefore, we made the first-order derivative curves to cancel this effect. The growth curve was similar to that in the control (water-treated) group, as shown in Fig. S13. The same trend was observed for CHX, although the slope was more stable (Fig. S14). Based on this experiment, the minimum inhibitory concentration (MIC)^77^ for the CHX PR_4_^+^ NPs and CHX PR_4_^+^ polymer NPs was determined to be 5.7 mM (Fig. S15). The MIC value of CHX was previously determined to be ≤2.47 M, according to Martins et al^78^. We calculated the molarity of the NPs based on the active component (CHX PR_4_^+^ NP) molar mass, which was determined to be 355 g mol^−1^, according to the mass spec results to make the comparison consistent.

In addition, to quantify cell death, a fluorescence live/dead assay was carried out for *S. mutans* in the planktonic stage. The results indicated that CHX PR_4_^+^ NPs and CHX PR_4_^+^ polymer NPs drastically reduced the number of live bacteria, while CHX NPs, CHX polymer NPs, and empty vesicles had comparably low dead cell percentages (Fig. 3b). This result agrees with that of the time-kill turbidity assay; therefore, CHX NPs and CHX polymer NP samples were excluded from the subsequent studies due to their ineffective antibacterial properties and instability. In addition, the empty vesicles antibacterial properties were negligible (Fig. 3b and Fig. S12), while their stability was not sufficient using the mentioned assembly method due to the lack of hydrophobic cargo. Of note, the statistically smaller number of live cells in the NP-treated vs. CHX drug-treated groups, was confirmed by two-way ANOVA (Fig. 3b and Supplementary Data 1).

The ROS (such as singlet oxygen, peroxides, and superoxides) are considered to be the major modulators of bacterial cell death in the presence of NPs. The ROS level in *S. mutans* was evaluated based on a green fluorescent probe, 2′,7′-dichlorofluorescein diacetate, which becomes intensely fluorescent when converted to 2′,7′-dichlorofluorescein in the presence of ROS (Fig. 3c). Based on the results, there is a large buildup of ROS in the CHX PR_4_^+^ polymer NP and CHX groups vs. the water-treated group. Notably, the ROS level in the CHX PR_4_^+^ polymer NP group is significantly higher than that of the positive control (CHX) group, confirming the efficacy of NPs in inducing cell death. The ROS generation was also observed in the mammalian NIH 3T3 cells with the increase of the NP concentration, which adversely affected the cell viability. The same is also observed in 3-(4, 5-dimethylthiazol-2-yl)-2, 5-diphenyltetrazolium bromide (MTT) assay. Hence, we adopted a lower concentration of the NPs during the in vivo studies, while extending the duration of the treatment (Fig. S16).

To explore the underlying mechanism, we investigated the disruption of the electrochemical gradient of the membrane, which is an indication of depolarization and cell death (Fig. 3d, e and Fig. S16). We utilized a membrane potential indicator dye, carbocyanine dye DiOC_2_(3) (3,3′-diethyloxacarbocyanine iodide), which fluoresces red in fully polarized bacteria and fluoresces green in depolarized cells^79^. The ratio of red:green fluorescence intensity is, therefore, a good indicator of cell polarization. Corrections were applied to account for the cell size and aggregation. The mean of the ratiometric parameter was calculated to be 467 for the control group and 204 for the NP treatment group with the distribution shown in Fig. S17, suggesting membrane damage and loss of cell polarization.

**Fig. 4:**
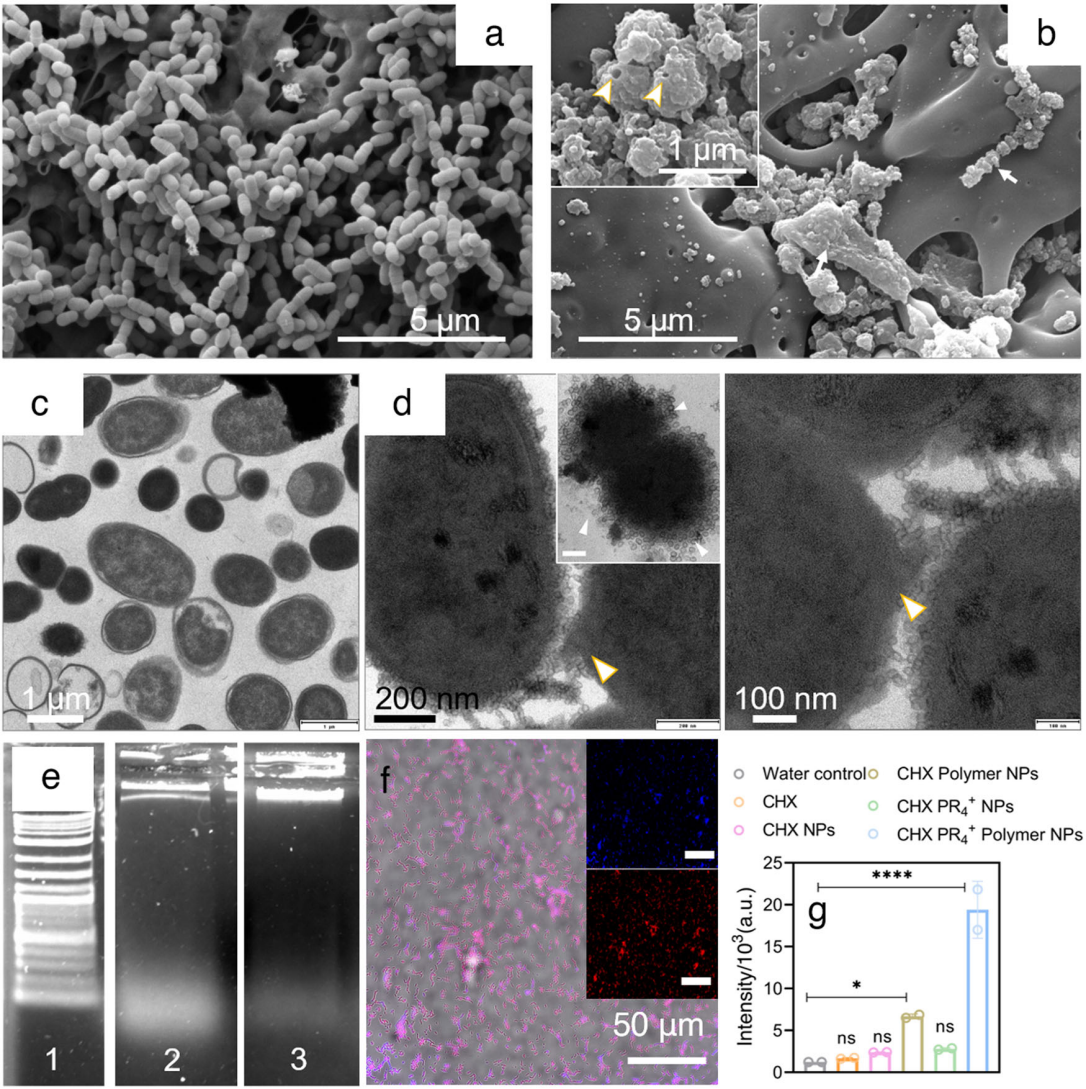
Antibacterial mechanism governing cell death. SEM images of planktonic *S. mutans* treated with **a** water and **b** CHX PR_4_^+^ polymer NPs with noticeable damage to the membrane and loss of cell morphology; arrows indicate the NPs attached to the bacterial membrane; TEM images of **c** control, water-treated, and **d** CHX PR_4_^+^ polymer NP-treated *S. mutans*. The arrow indicates the damaged membrane, and the arrow in the inset figure indicates the NPs on the bacterial outer membrane; **e** downstream damage to *S. mutans* as a result of treatment with NPs as investigated with a DNA gel electrophoresis assay, 1: ladder, 2: CHX, and 3: CHX PR_4_^+^ polymer NP treatment; **f** confocal images from a TUNEL assay of CHX PR_4_^+^ polymer NP treatment, scale bar 50 μm. All the scale bars are 50 µm. Blue is the nuclear stain, and red indicates the TUNEL-positive cells. **g** The TUNEL assay red channel intensity as measured by plate reader. Ordinary one-way ANOVA w.r.t. water, *p* values < 0.1 and 0.0001 are indicated by * and ****, respectively. Error bar represents mean ± SD.

To further unravel the underlying antibacterial mechanism, an examination of the cellular morphology and membrane integrity upon treatment with the NPs was performed using scanning electron microscopy (SEM; Fig. 4a, b) and TEM (Fig. 4c, d). The control (water-treated) group showed cocci with evenly distributed cytoplasmic content and no noticeable damage to the cell membrane (Fig. 4a, c). In contrast, the NP-treated group (CHX PR_4_^+^ polymer NPs) showed remarkable damage to the membrane with a large number of the NPs latched onto the membrane, which is consistent with both the SEM (Fig. 4b) and TEM images (Fig. 4d and Fig. S18).

Most of the cells were severely deformed to the point of complete collapse. The strong interactions between the NPs and the cellular membrane could have originated from the electrostatic interactions between the super-cationic NPs with the negatively charged peptidoglycans of the gram-positive *S. mutans*. Phosphonium ions are expected to have a more potent antibacterial effect, presumably due to the larger ionic radius of the P ion and higher ionization effect, which can lead to easier association with the negatively charged bacterial membrane^52^. These interactions could result in the rupture of the bacterial cell membrane, leading to the leakage of the intracellular content (e.g., DNA and RNA), which would ultimately hinder bacterial function.

Further mechanistic studies at the subcellular level were carried out to clarify the functions of the NPs in inhibiting the bacteria. Initially, the interactions of the plasmid DNA (pDNA, pBR322) with the CHX PR_4_^+^ polymer NPs and CHX were investigated after 1 h of exposure at various concentrations of the treatment system (5.7, 1.4, 0.35, and 0.09 mM; Fig. S19). Here, water treatment was considered as the negative control. Interestingly, at 5.7 mM concentration of NPs, pDNA was completely degraded and no band was observed. At lower concentrations, this effect is suppressed, although trailing from fragmentation can still be observed; CHX showed sharp bands, and the pattern is more similar to that of the untreated sample for pDNA experiment.

Next, *S. mutans* was treated with NPs, and then genomic DNA was extracted (Fig. 4e). The gel electrophoresis results indicated that the DNA fragments traveled beyond the DNA ladder due to their low molecular weight, confirming the severe degradation of the DNA by the NPs (Fig. S20). We also tested the DNA damage by a terminal deoxynucleotidyl transferase dUTP nick-end labeling (TUNEL) assay (Fig. 4f, g). The confocal images are presented in Fig. 4f, where red (Tunnelyte™ Red) fluorescence indicates a nick in the DNA and blue (Hoechst) indicates the nuclei (used to determine the number of cells). The analysis of the ratio of red: blue intensity revealed that CHX PR_4_^+^ polymer NP induced apoptosis in *S. mutans* (*p* value = 0.001 vs. water group, one-way ANOVA; Fig. 4g and Supplementary Data 1). Collectively, these results demonstrated the success of CHX PR_4_^+^ polymer NPs in fragmentation and bacterial cell growth inhibition, even at the subcellular level.

### Polymer-wrapped therapeutic nanoparticles inhibit and disperse biofilms in vitro and ex vivo

**Fig. 5:**
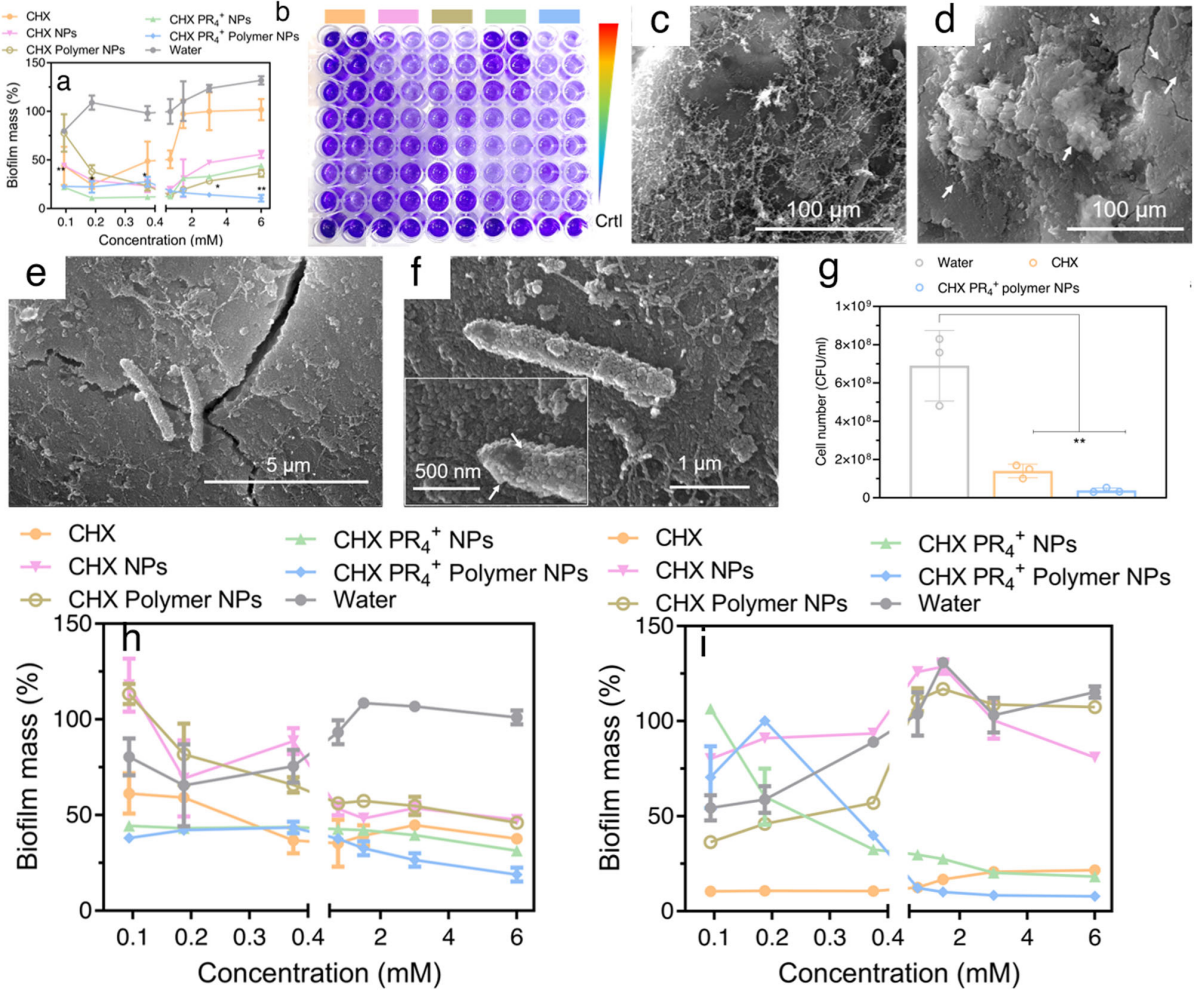
Antibiofilm properties of CHX PR_4_^+^ polymer NPs against mature biofilms (48-h old). Biofilm **a** dispersion and **b** the corresponding plate digital photographs of the wells; the top-most well is the control with the colors on the top representing sample ID, the wedge gradient indicates decreasing concentrations. Here, two-way ANOVA was performed, and *p* values < 0.1, and 0.01 are indicated by * and **, respectively. Biofilm and EPS inhibition for biofilms grown on human tooth **c** control (water-treated) and **d** NP-treated samples. The images were acquired at the same magnification; **e** few surviving bacteria in the treated sample and **f** the attachment of the NPs to the bacteria are represented in the higher magnification and pointed by arrows; **g** the colony count assay from experiment (**g**), *n* = 3, ordinary one-way ANOVA, *p* value < 0.01 is indicated with **, biofilm **h** dispersion and **i** inhibition as measured by the fluorescent resazurin assay. Error bar represents mean ± SD.

Having established the efficacy of the antibacterial CHX PR_4_^+^ polymer NPs against the planktonic form of *S. mutans*, we sought to understand the antibiofilm properties of NPs because the elimination of the biofilm is more difficult due to the shielding effect of the EPS. The NP-mediated biofilm dispersion was investigated in an established 48-h-old *S. mutans* biofilm. The results from crystal violet staining^80,81^ confirmed the dose-dependent and effective dispersal of the biofilm mass compared to the negative control (Fig. 5a and Supplementary Data 1). This result was further corroborated by resazurin assay as inidicated in **Fig. 1h** that targets the metabolic activity of the cells and releveals the viability. Furthermore, the ability of the NPs to inhibit the biofilm was explored by preincubating the bacteria with the NPs during the bacterial biofilm formation stage (Fig. 5i). Remarkably, the biofilm was inhibited by more than 90% at 0.7, 1.4, 2.8, and 5.7 mM in the NP-treated groups, suggesting that the NPs had an excellent inhibitory effect on biofilm formation. As mentioned, the molarity is calculated based on the results from mass spectroscopy.

Subsequently, an ex vivo model of dental biofilm was developed on extracted human teeth. Briefly, a mature biofilm was grown on human molars placed on agarose (to mimic the soft tissue) for two consecutive days, while the teeth were placed sideway and dipped fully in the broth (Fig. S21). This model is based on our previous work^77^. Subsequently, the teeth were treated with the NPs for 4 h (fully dipped in treatment suspension), and then were gently sonicated for a few minutes to obtain cultivable bacteria for use in a colony counting assay. As shown in Fig. 5f, the CHX PR_4_^+^ polymer NP group showed a significantly lower number of cultivable bacteria relative to that of the water-treated group, highlighting the outstanding antibiofilm properties of the NPs in the ex vivo model of the human tooth as was corroborated by the resazurin  and the ex vivo assays.

### Degradation of therapeutic CDot NPs in simulated saliva

**Fig. 6:**
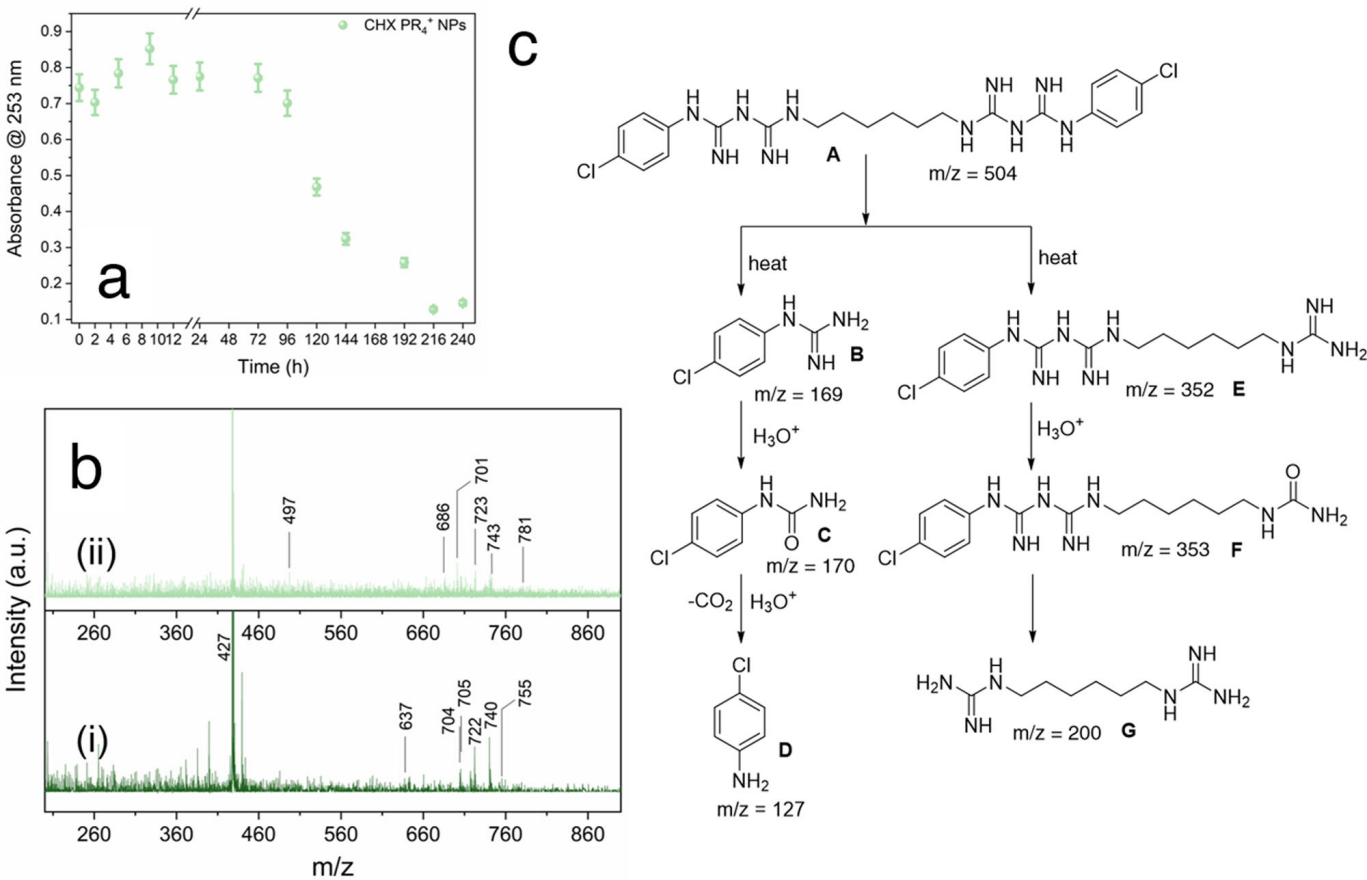
Biodegradation of CHX PR_4_^+^ NPs in artificial saliva tracked for consecutive 10 days. **a** The measured absorbance of CHX PR_4_^+^ NPs at 253 nm; **b** mass spectra of (i) CHX PR_4_^+^ NPs in saliva and (ii) degraded CHX PR_4_^+^ NPs; **c** the proposed scheme for the degradation pathway concluded from the mass spectrometry results.

Although several antibacterial NPs based on organic and inorganic materials have been reported with good functional properties, their lack of biodegradability is a major concern to fully realize their translational potential^82^. We investigated the fate of the developed CHX PR_4_^+^ NPs in vitro by exposing them to the simulated saliva for medical and dental research. Subsequently, the absorbance of the NPs was screened as depicted in Fig. 6a and Fig. S21. A decreasing trend in the absorbance was noticed in the course of experiment (i.e., 10 days) indicating the degradation of the NPs (Fig. S22). We sought further confirmation for the mechanism of degradation of the collected samples by conducting MS analysis in electrospray ionization (ESI) mode (Fig. 6b and Figs. S23–26). A plausible scheme for the enzymatic degradation of CHX (A) after carbonization is proposed (Fig. 6c) to involve the formation of various fragmented products B–G. We presume that CHX PR_4_^+^ NPs could have been degraded to form guanidine B and E followed by hydrolysis to generate urea C and F. Amine D and guanidine G are formed by hydrolysis followed by decarboxylation reaction and heating, respectively.

From ESI-MS measurements, the products corresponding to the mass to charge (*m*/*z*) values of 427, 637, 704, 705, 722, 740, 741, and 755 are observed at the initial time point of degradation, indicating the formation of intermediates highlighted in C–G as in Fig. 6c and Supplementary Data 1. After 1 day and 2 days degradation by enzyme, new products corresponding to *m*/*z* values of 680, 781 and 497, 685, 772, respectively are identified, indicative of D–G. Over the 8 days incubation period, CHX PR_4_^+^ NPs further degraded while several new mass peaks appeared at *m*/*z* values of 686, 701, 723, and 743, which have been assigned to intermediates C, D, F, and G. The degradation studies continued over the course of 10 days and a few additional peaks were observed corresponding to *m*/*z* values of 773 and 828, indicating the formation of C, D, and G intermediates. Overall, these results suggest that should the therapeutic CDots not be cleared upon topical application, they can be degraded to smaller metabolites as identified above.

### Polymer-wrapped therapeutic nanoparticles suppressed the biofilm in vivo

**Fig. 7:**
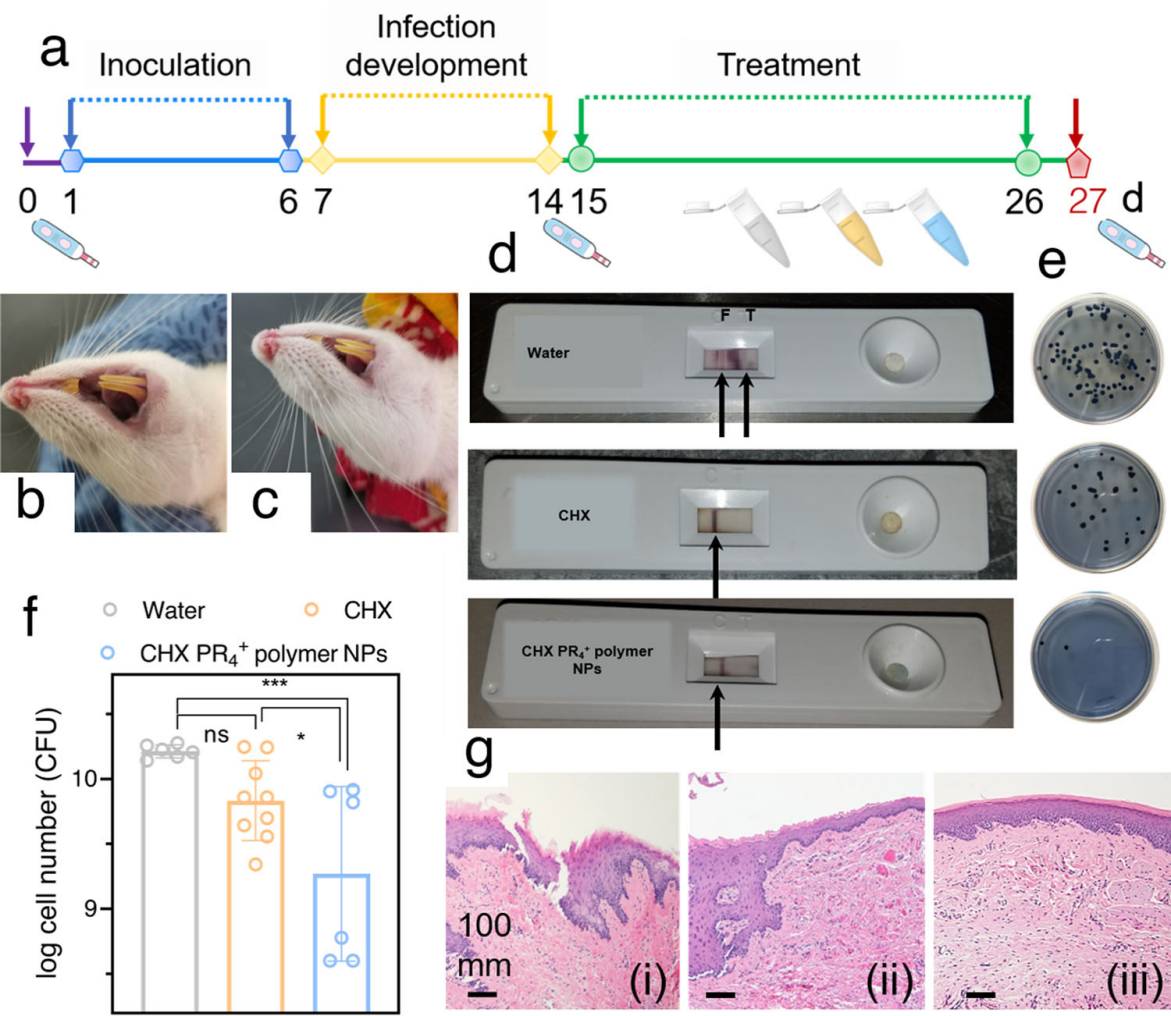
The in vivo efficacy of the NPs in a rat model of the dental biofilm. **a** Timeline of the in vivo animal treatment, the treatments (water, CHX, and CHX PR_4_^+^ polymer NPs) are represented in the centrifuge tubes; the dental hygiene in the animal model **b** before treatment and **c** after treatment with NPs; **d** the outcomes of the in vivo *S. mutans* detection kit; the results were positive for the water-treated group and negative for CHX- and CHX PR_4_^+^ polymer NP-treated groups; **e** the cultivable bacteria collected from the harvested animals’ teeth. Bacitracin-modified mitis salivarius agar plates, which can specifically grow *S. mutans*, have been employed here. **f** The collective results from the plate count assay of the cultivable bacteria after obtaining their jaws (*n* = 3 biological replicates); ordinary one-way ANOVA, *p* value < 0.1 and 0.001 are indicated by * and ***, respectively. Error bar represents mean ± SD. **g** H&E histological analyses of gingiva for (i) control, (ii) CHX, and (iii) CHX PR_4_^+^ polymer NP groups. The scale bars are all indicating 100 nm.

To validate the antibiofilm effect in vivo, we created biofilm virulence in a rodent model of dental biofilm according to the plan indicated in Fig. 7a based on our previous rat model of dental biofilm^48^. Prior to the infection, Sprague Dawley rats were determined to be *S. mutans* free with an *S. mutans* detection kit, and then the rats were randomly divided into three groups, namely, the water, CHX, and CHX PR_4_^+^ polymer NP treatment groups. *S. mutans* was inoculated on the rat’s incisors on six consecutive days, and then seven days were allowed for the induction of the biofilm. At the end of this period, the infection by *S. mutans* was confirmed for all animals using a detection kit. Daily regimen with the formulation (1.4 mM, 100 μl) was maintained for 11 days, which involved a brief 1 min exposure once per day and then treatment at 2.3 mM, 100 μl for the last 2 days. No loss of animals occurred during the experiment, and the animals equally gained weight without significant differences between the groups (*p* > 0.05).

An improvement in dental hygiene was observed after treatment with the NPs (Fig. 7b, c); although a better determinant of the dental hygiene was required for further confirmation of the visual inspection. Hence, before sacrificing the animals, the presence of *S. mutans* was determined using a lateral flow assay device. The assay provides a positive or negative result based on the presence or absence of detectable *S. mutans* (Fig. 7d). Interestingly, the kit gave a negative result for the CHX PR_4_^+^ polymer NP-treated animals, which was comparable to that achieved with CHX; however, for the water-treated group, the kit provided a positive result. The teeth were harvested from the mandible, and then the extracted bacteria were inoculated on mitis salivarius-bacitracin (MSB) agar plates (selective for *S. mutans* growth) to count the cultivable colonies (Fig. 7f). The CHX PR_4_^+^ polymer NPs were substantially better at eradicating the dental biofilm relative to the negative control (*p* value = 0.0008, Supplementary Data 1). Notably, the performance of CHX PR_4_^+^polymer NPs was superior to that of CHX (*p* value with respect to (w.r.t.) water = 0.0308). For comparison purposes, representative plates are presented in Fig. 7e. To assess the safety and biocompatibility of the NPs, histopathological evaluations on the major organs (Fig. S27) and the gingiva (Fig. 7g) were conducted by a board-certified histopathologist. For the water-treated group, part of mucosal epithelium was hyperplastic and hyperkeratotic with hints of pink, white, and blue layering; the sub epithelium was slightly hyperemic, and the severity of the lesion was mild (+). On the other hand, the severity of the lesion in the CHX-treated group was either mild (+) or minimal (±). Finally, the severity of the lesion for the CHX PR_4_^+^ polymer NP group was either minimal (±) or none. No significant differences were observed in the major organs, including hearts, lungs, livers, kidneys, spleens, and intestines, among the animals across all the groups (Fig. S27).

### Polymer-wrapped therapeutic nanoparticles maintained the ecological balance of the microbiota in vivo

The human oral cavity is estimated to harbor over 700 different species of bacteria. A vast majority of them promote oral health by interacting with the immune system and inhibiting the colonization of pathogenic species^83^. Hence, to achieve the maximum efficiency, a treatment needs to modulate the pathogenic bacteria in a targeted manner, while sparing most of the healthy microbiome^28^. Furthermore, it is being demonstrated that a community of multiple acidogenic and acid-tolerant species as a group are responsible for biofilm development^84^. For example, studies have shown the association of *S. mutans* and *Lactobacillus spp*. with childhood caries^85^. Other bacterial species found in cariogenic biofilms include *Streptococcus. salivarius*, *Streptococcus sobrinus*, and *Streptococcus parasanguinis*^13^, suggesting that the holistic targeting of acidogenic bacteria would be a promising strategy.

**Fig. 8:**
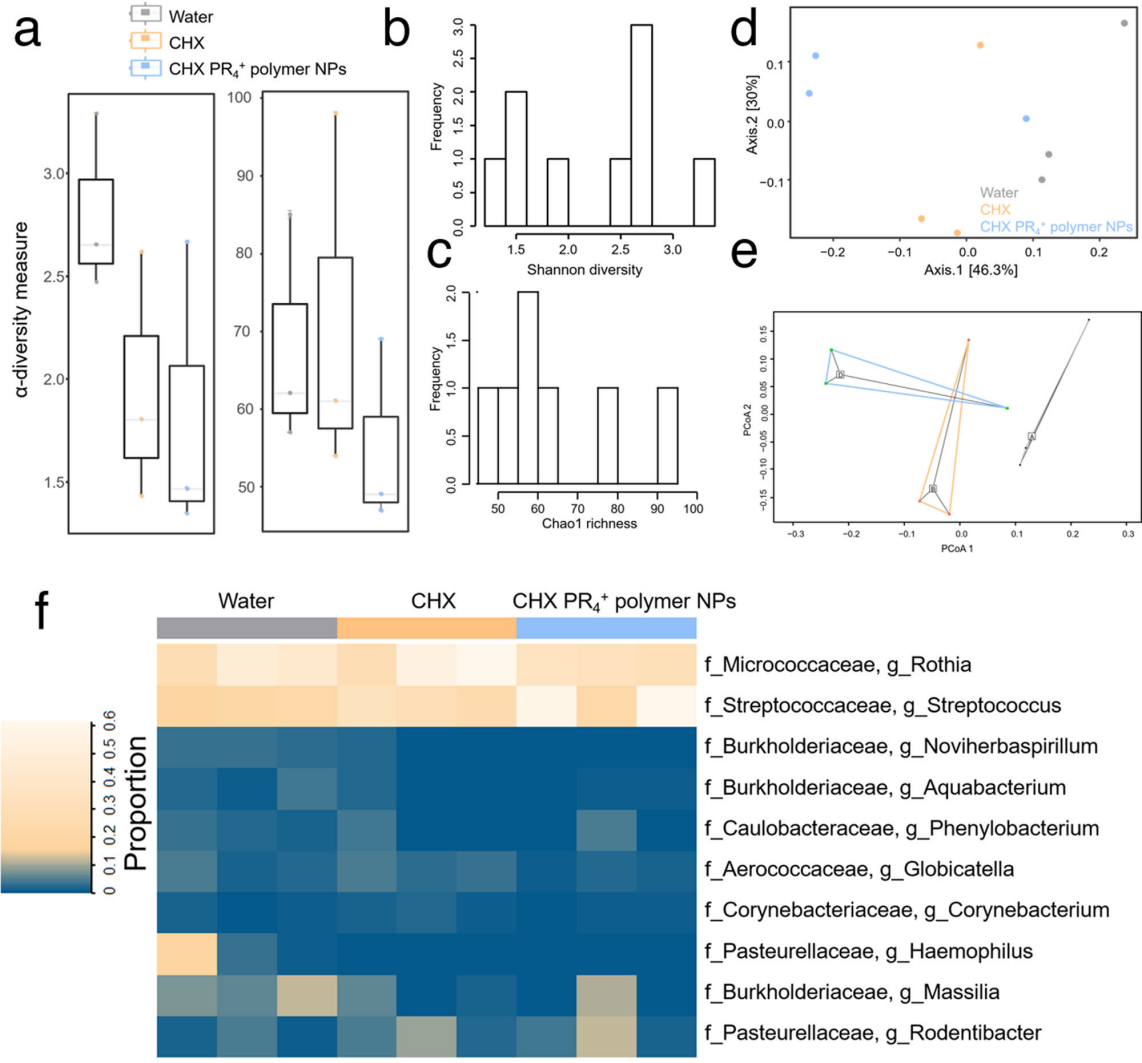
The assessment of the oral microflora of the rats (*n* = 3 per group) treated with various formulations. **a** Shannon α-diversity index and Chao 1 richness index for different groups; **b** Shannon α-diversity index and **c** Chao 1 richness normal distribution test; **d** PCA ordination plot of the oral microbiota in various groups, where the distance between each point indicates the difference between those samples; **e** Bray–Curtis principal coordinate analysis (PCoA), which indicates the β-diversity in each group; the analysis revealed that there are no significant differences among the groups based on their Bray–Curtis distances, and the PERMANOVA and ANOSIM tests have *p* value > 0.05 and in both cases; **f** the heatmap indicates the bacterial taxa found in all samples according to treatment type, and there are no significant differences in the proportions of each genus among the groups.

To elucidate the impact of the NP treatment on the dental microbiota, microbial swabs were collected from rat incisors at the end of the 13-day topical treatment period, and DNA was isolated. Using the Illumina MiSeq platform, sequencing targeting the V3 and V4 regions of the 16S rRNA gene was performed, and the sequences were analyzed for bacterial composition and abundance (Fig. 8 and Figs. S28–30). α-Diversity comparisons (i.e., the microbial diversity within each sample) for bacterial richness across treatment groups were performed using standard ANOVA, and no significant differences across groups were observed (Fig. 8a, online data repository); *p* = 0.204, Shannon diversity index (Fig. 8b) and *p* = 0.341, Chao 1 richness index (Fig. 8c), implying that neither the diversity nor the richness of the treatment group were affected. In addition, β-diversity analysis was performed using Bray–Curtis distance (Fig. 8d), which similarly showed no differences between treatment groups when compared using PERMANOVA (Fig. 8e)^86^ (*p* = 0.16) and ANOSIM^67^ (*p* = 0.294). The analysis of the overall proportion of the microbiota composition (Fig. 8f) did not reveal any significant differences among the groups. Therefore, it can be concluded that our NPs are strategically designed to be pH responsive and effectively kill cariogenic bacteria, while not upsetting the ecological balance of the dental microbiome. Targeting the cariogenic bacteria selectively either by active or passive approaches is of primary research interest among the microbiologists^87–89^. Herein, we have developed a novel bio-engineering approach which generated an antimicrobial agent that becomes active only in the low pH microenvironments of biofilms, and thus passively targets the cariogenic bacteria utilizing the physical characteristics of the biofilm.

The design and selection of NPs for achieving efficient antimicrobial activity for the eradication of biofilms must exploit the physical characteristics of the biofilm. We reported for the first time a new class of “particle-in-particle” agents that possess multiple copies of antibiofilm CDots to treat *S. mutans* biofilms both in vitro and in vivo. This strategy utilized the pathological pH of a biofilm, which not only led to biofilm dispersal and enhanced bacterial susceptibility, but also allowed in situ antibacterial properties without requiring an external drug. Moreover, unlike the widely applied strategies, no external stimuli (e.g., H_2_O_2_ and photo-stimulation) were utilized, which helps minimize unintentional toxicity.

We envision that the high surface functional group density of the self-assembled construct will enable the future introduction of targeting moieties via facile chemistry to tailor these particles for combating specific pathological bacteria. This approach would concomitantly be applied in the sensitive detection and molecular imaging of biofilms using the luminescence of CDots. We envisage that these NPs can be applied topically in the form of a paste, as was demonstrated in this study, to improve patient compliance with the product.

Moreover, in an era where antibacterial resistance is a challenge, the development of new antibiotic-free approaches with superior properties compared to their gold standard clinical counterparts has broader implications for global health. Here, these NPs did not disturb the ecological balance of the microbiota, which may indicate a lack of resistance in the species.

Finally, we believe that these NPs can open platforms for treating more vicious infective diseases caused by biofilms, such as infective endocarditis, otitis media, urinary tract infections, and implant-associated biofilms. Considering the biodegradable, tunable, and self-contained nature of these NPs, these milestones do not seem far-fetched.

## Methods

### Materials

All the starting materials for the synthesis were purchased from Sigma Aldrich (St. Louis, MO, US) unless noted otherwise. Nano pure water (0.2 × 10^−6^ m, 18 MΩ cm) was used throughout the experiments. A culture tube of *S. mutans* was bought from VWR International (Chicago, IL, US) and was subcultured in either BHI broth or BHI agar, which were both obtained from Becton Dickinson (Franklin Lakes, NJ, US).

### Nanoparticle synthesis and characterization

CHX NPs and CHX PR_4_^+^ NPs were prepared using a hydrothermal method. In a typical process, 300 mg of CHX (CAS no. 55-56-1) or 210 mg of CHX and 90 mg of tributylhexadecylphosphonium bromide (CAS no. 14937-45-2) were dissolved in 30 ml of methanol, and then subjected to heat treatment in an autoclave synthesizer at 180 °C for 24 h. The as-synthesized CDots were sonicated for 5 min using a probe sonication instrument (Q700, Qsonica Sonicators, Newtown, CT) at Pulsed Amp, 1; 2 s on, and 1 s off. The CDots were passed through syringe filters with mesh sizes of 0.45 and 0.22 μm (Biomed Scientific), respectively. Subsequently, the solvent was evaporated on a rotary evaporator, and the sample was allowed to dry overnight in a vacuum oven to remove any remaining methanol. For the synthesis of the CHX NPs and CHX PR_4_^+^ NP, 100 mg of the above CDots were dispersed in 8 ml of water and tip sonicated for 15 min to yield a NP dispersion. At this stage, it was noticed that the CDots (especially the CHX NPs) did not have desirable water dispersibility and formed oily suspensions due to the hydrophobicity of the CDots from the CHX precursors. To prepare the CHX polymer NPs and CHX PR_4_^+^ polymer NPs, additional steps were taken to wrap the NPs in the pH-responsive shell. PS-*b*-PDMA (*M*_*n*_ × 10^3^ = 6.7-*b*-9.2, PDI = 1.06) (100 mg) procured from Polymer Source, Inc. (Quebec, Canada) and 100 mg of CHX NPs or CHX PR_4_^+^ NPs were dispersed in 2 ml of CHCl_3_ and sonicated for 5 min on ice in a test tube. Then, the CHCl_3_ was slowly evaporated under a gentle stream of nitrogen while rotating the tube rapidly to yield a thin layer of materials in the tube. An oil diffusion pump was utilized to remove any residual organic solvent (2 h). To further ensure the absence of any organic solvent, the sample was dried overnight in a vacuum oven. The samples were then redispersed in 8 ml of water (to a concentration of 12.5 mg ml^−1^ or 28.5 mM of CDots) and sonicated for 15 min, as detailed above. A similar procedure was adopted for the formation of empty micelles; however, the sample was sonicated for an additional 8 min (23 min total) to yield stable particles. The samples were stored in the fridge until further use to maintain their stability and were sonicated and vortexed briefly before each experiment.

The samples were extensively characterized to determine their physicochemical properties. The morphology of the NPs was inspected by TEM (FEI Company, Oregon, US), using an instrument equipped with a Peltiercooled Tietz (TVIPS) 2*k* × 2*k* charge-coupled device camera. The samples were drop-deposited on a carbon-coated copper grid (TED PELLA Inc., Redding, CA, US), and then the excess liquid was wicked away with filter paper. The samples were negatively stained with a 2% solution of uranyl acetate. For AFM imaging, the samples were diluted and then drop-cast on a mica-attached to steel disk before image acquisition using a Bruker MultiMode Nanoscope IIIA (Billerica, MA, US).

For further photophysical characterization, absorbance spectroscopy was performed using a GENESYS 10 UV–vis spectrophotometer (Thermo Scientific, MA, US). The emission spectra were collected on an Infinite 200 PRO multimode microplate reader (Tecan, NC, US), and after adjusting the concentration of CDots to 2.5 mg ml^−1^, excitations at 320, 370, 420, 470, and 520 nm were observed.

For the chemical characterizations, initially, the sample was analyzed by MS in ESI mode at the MS facilities at UIUC. The ^1^H NMR, ^13^C NMR, and ^31^P NMR spectra of the samples were acquired on a multinuclear, 500-MHz Ultrashield Plus (Bruker Daltonics, Inc., Billerica, MA, USA) system equipped with a 5 mm CryoProbe (Bruker, Model CB500) in CDCl_3_ (Cambridge Isotope Laboratories, Inc., Cambridge, MA). The results were analyzed using MestRenova 11.0 software (Mestrelab Research SL; Santiago de Compostela, Spain). Moreover, to prepare the samples for Fourier transform infrared (FTIR) spectroscopy, they were dried on MirrIR IR-reflective glass slides (Kevley Technologies, Chesterland, OH, USA), and their transmission was subsequently recorded in attenuated total reflectance mode on a Nicolet Nexus 670 FTIR instrument (ThermoFisher scientific, Waltham, MA, US). XPS was conducted on small piece of sample on glass slides that had been dried under vacuum overnight. A Physical Electronics PHI 5400 spectrometer using Al Kα (1486.6 eV) radiation was utilized for acquiring the spectra. CasaXPS software was used for peak decomposition and referencing to the adventitious C–C bond (284.8 eV)^90^.

The concentration of the NPs and their hydrodynamic diameter were measured by ZetaView system (ParticleMetrix, GmbH) for a sample of 2.5 mg ml^−1^ of CHX PR_4_^+^ polymer NPs. The following formula^91^ was applied assuming the CHX PR_4_^+^ molecular mass to be 355 mg ml^−1^ based on mass spec data:

1 $${{\mathrm{avg}}\;{\mathrm{particle}}\;{\mathrm{mass}}}\left({\mathrm{{mg}}}\right)=\frac{{{\mathrm{incorporated}}\;{\mathrm{CHX}}\;{{\mathrm{PR}}}}_{4}^{+}\;{\mathrm{{NPs}}}\;\left({\mathrm{{mg}}\cdot{{\mathrm{ml}}}}^{-1}\right)}{{{\mathrm{CHX}}\;{\mathrm{PR}}}_{4}^{+}{{\mathrm{polymer}}\;{\mathrm{NPs}}\;{\mathrm{particle}}\;{\mathrm{concentration}}}\;\left({{{\mathrm{ml}}}}^{-1}\right)}$$

2 $${\mathrm{{no}}.\;{\mathrm{of}}\;{\mathrm{CHX}}\;{\mathrm{PR}}}_{4}^{+}\;{{\mathrm{per}}\;{\mathrm{CHX}}\;{\mathrm{PR}}}_{4}^{+}\;{{\mathrm{polymer}}\;{\mathrm{NPs}}}= 	\frac{{{\mathrm{avg}}\;{\mathrm{particle}}\;{\mathrm{mass}}}\left({{\mathrm{mg}}}\right)}{{{\mathrm{CHX}}\;{\mathrm{PR}}}_{4}^{+}\;{{\mathrm{molecular}}\,{\mathrm{wt}}}\,\left({{\mathrm{mg}}\cdot{{\mathrm{ml}}}}^{-1}\right)}\\ 	\times 6.022\times {10}^{23}\left({\mathrm{mol}}^{-1}\right)$$

### pH-Dependent nanoparticle release

CHX PR_4_^+^ polymer NPs were exposed to pH = 7.3 (PBS buffer) and pH = 4.5 (acetate buffer; sink conditions, 37 °C) through a dialysis membrane (MWCO = 10,000 Da). At specific time points, 2 ml of the dialysate was taken for UV–vis spectroscopy, and 2 ml of buffer was added to maintain the concentration (1.59 µM in 500 ml buffer). The collected samples were concentrated in SpeedVac system. The calibration curve of the known NP concentration (Fig. S11) was utilized to calculate the cumulative percent release.

### Degradation of the nanoparticles with artificial saliva

The ability of CHX PR_4_^+^ NPs to undergo biodegradation by artificial saliva was investigated by incubating 5.7 mM NPs (700 µl) in 2800 µl of artificial saliva (Artificial Saliva for Medical and Dental Research, Pickering Laboratories, CA) at 37 °C. The artificial saliva contained 10 g l^−1^ sodium carboxymethyl cellulose at pH 6.8. Samples (100 μl) were collected at predetermined time points. UV−vis and MALDI analyses were used to characterize the biodegradation of the CDots.

### In vitro antibacterial assessment

#### Microdilution turbidity assay

The turbidity assay used the absorbance of the bacteria at 600 nm (OD_600_) as an indication of the concentration of bacteria. *S. mutans* was grown in BHI broth overnight under anaerobic conditions in a GasPak™ Container System (Becton Dickinson, Franklin Lakes, NJ, US) in a 37 °C chamber containing 5% CO_2_ until the OD_600_ = 0.6 (mid-exponential phase). The bacteria were subcultured to OD_600_ = 0.4 and then treated with NPs (5.7 mM) for 4 h. They were then immediately transferred to a 96-well plate in triplicate, and the plate was moved to a plate reader (BioTek Synergy HT, US) with constant shaking at 37 °C. The absorbance of each well was read every 30 min. The MIC was defined as the minimal concentration that completely inhibits the bacterial growth. The absorbance results were used to calculate the cell viability, and then the MIC was obtained based on Lambert and Pearson’s model^92^.

#### Live/dead bacterial viability assay

A SYTO9/PI (propidium iodide) double staining kit, namely, LIVE/DEAD® BacLight™ Bacterial Viability Kit (L7012, ThermoFisher Scientific, Grand Island, NY, USA) was used. The assay was conducted based on the procedure described by the manufacturer. The OD of *S. mutans* grown to the mid-log phase was adjusted to 0.3, and then 800 μl of the bacteria suspension was exposed to various treatment formulations at 5.7, 2.8, 1.4, and 0.7 mM for 4 h. The samples were then washed thoroughly twice with Tris-HCl buffer to remove the nonspecific background contaminants. *S. mutans* was resuspended in 400 μl of Tris-HCl and 400 μl of the dye mixture (54 μl of PI and 54 μl of SYTO^TM^ 9 in 9892 μl of DI water) in the dark for 15 min. The fluorescence of the bacteria was read on a plate reader at *λ*_excitation_/*λ*_emission_ = 480/500 and 490/635 nm. Ten microliters of the above mixture were layered between a cover slip and glass slide for visualization on a Leica SP8 UV/Visible Laser Confocal Microscope (Leica Microsystems, Germany).

#### Scanning electron microscopy of the planktonic bacteria

*S. mutans* with OD_600_ was treated with 5.7 mM CHX PR_4_^+^ polymer NPs or an equivalent volume of water and then filtered through a syringe filter to collect the bacteria (pore size = 0.45 µm). The filter membranes were separated and fixed with EM-grade fixative using 2.0% paraformaldehyde and 2.5% glutaraldehyde in 0.1 M Na-cacodylate buffer (pH 7.4) on ice for 2 h. The cells were then dried using a graded ethanol series (37, 67, 95, and 100% ethanol 3×) for 10 min each, and then they were subjected to critical point drying (Autosamdri®-931, Rockville, MD). SEM images were acquired on Au/Pd sputtered samples using an XL30 ESEM-FEG Field-Emission Environmental SEM instrument (Philips/FEI, Hillsboro, OR).

#### Transmission electron microscopy of the planktonic bacteria

*S. mutans* with OD_600_ was treated with 5.7 mM CHX PR_4_^+^ polymer NPs or an equivalent volume of water and was washed with EM-grade buffer once before the addition of EM-grade fixation solution (2% paraformaldehyde, 2.5% glutaraldehyde in 0.1 M Na-cacodylate buffer). The samples were fixed for 2 h on ice and then washed with 0.1 M Na-cacodylate buffer. Post-fixation in osmium tetroxides, en bloc in uranyl acetate, dehydration, infiltration, and growth were sequentially conducted before ultrathin sectioning with an ultramicrotome (Ultracut UCT, Leica, IL, US).

#### Membrane potential analysis

A BacLight Bacterial Membrane Potential Kit purchased from Invitrogen was used to determine if the polarity of the cells was preserved during treatment. This kit contains carbocyanine dye, DiOC2(3), which is a green fluorescent dye (*λ*_ex_ = 488 nm), and its emission shifts toward red at the higher cytosolic concentrations caused by larger membrane potentials. The treatment was carried out as usual, in which *S. mutans* with OD_600_ = 0.6 was treated with 5.7 mM CHX PR_4_^+^ polymer NPs or an equivalent volume of water for 4 h. The control nontreated sample was then used to calibrate the cell concentration to ~10^6^ cells ml^−1^. Then, 10 µl of DiOC2(3) was added, and the mixture was incubated for 30 min. Flow cytometry was conducted in the FITC (green) and Texas red dye (red) channels.

#### Laddering assay of the DNA

A further subcellular investigation was achieved with the aforementioned assays. Initially, 10 ng μl^−1^ of pBR322 vector DNA (pDNA) was exposed to 5.7, 1.4, 0.35, and 0.09 mM CHX or CHX PR_4_^+^ polymer NPs for 60 min. Then, 5 μl of the loading dye was added. A 2 wt% agarose gel was run for 30 min at 120 mV in 1× Tris-borate EDTA buffer at pH 8.0 (ref. ^74^). The gel was then immersed in 3% ethidium bromide solution (10 mg ml^−1^) for 5 min, washed with ethidium bromide solution for another 5 min, and imaged on a gel doc system (Bio-Rad, Hercules, CA, USA). A 1 kb DNA ladder (TrackIt™, Invitrogen, Carlsbad, CA, USA) and the water-treated sample were used as controls.

In the following step, the genomic DNA from *S. mutans* treated with 5.7 mM of various formulations for 4 h and was extracted utilizing the PurElute™ Bacterial Genomic Kit (EdgeBio, Gaithersburg, MD, USA). Subsequently, 25 μl of the extracted DNA (in Tris-HCl) and 5 μl of the loading dye were used for gel electrophoresis.

#### TUNEL assay

A Cell Meter™ TUNEL apoptosis assay kit (red fluorescence) was purchased from AAT Bioquest, Inc., CA, USA, and was used following the procedure provided by the manufacturer. Briefly, 800 µl of *S. mutans* (OD_600_ = 0.6) was treated with various formulations at 5.7 mM for 4 h. The samples were then centrifuged, and the supernatants were discarded. The cells were fixed with 400 µl of 4 wt% formaldehyde (prepared in DPBS) for 30 min at room temperature. To enhance cell permeability, 400 µl of 0.1 wt% Tween 20 was added for 5 min, and then the bacteria were washed three times with DPBS. Reaction buffer (200 µl) and 2 µl of 100X Tunnelyte™ Red were added to each sample. The solutions were stored at 37 °C in the dark for 60 min and then washed three to five times with DPBS. The nuclei were stained with 1× Hoechst, and the samples were imaged with CLSM at *E*_*x*_/*E*_*m*_ = 350/460 nm and 550/590–650 nm. The data were analyzed with Fiji software (NIH, Bethesda, MD, USA) by splitting the red and blue channels, and using the Find Maxima function^93^.

#### MTT assay

NIH 3T3 cell line was cultured in Dulbecco’s modified Eagle’s medium with high glucose containing 10% fetal bovine serum and antibiotic, pen strep (100 units ml^−1^ penicillin and 100 µg^−1^ ml^−1^ streptomycin). Cells were incubated at 37 °C with a CO_2_ level of 5% in humidified atmosphere (relative humidity >95%). Trypsinization using 0.25% trypsin–EDTA was followed for passaging of cells at regular intervals. Cytotoxicity of the particles at different concentrations were evaluated by conventional MTT assay. In a typical experiment, cells were seeded at the density 10,000 cells per well in a 96-well microplate. After 24 h, cells were treated with the particles of different concentrations for 4 h followed by replacement with 200 µl of fresh medium and incubation for another 20 h. At the end, each well was treated with MTT (20 µl, 5 mg ml^−1^ in DPBS) and incubated for 4 h. Finally, whole medium was removed from the wells, DMSO (200 µl) was added and absorbance was read on a microplate reader. Experiments were performed in triplicates for each concentration and the results expressed are from three such independent experiments.

### Evaluation of in vitro and ex vivo antibiofilm properties

#### Inhibition and dispersion assays

Both inhibition and dispersion assays were developed. For the dispersion assay, *S. mutans* was adjusted to an OD_600_ of 0.6. A total of 200 µl of the bacterial suspension was seeded in a 96-well sterile plate for 48 h to form mature biofilms. The biofilms were gently washed once with Tris-HCl buffer and then treated with various concentrations of CHX PR_4_^+^ polymer NPs for 4 h. Notably, the experiment with CHX was impeded due to its poor water solubility. The plates were then washed with PBS and treated either with crystal violet or resazurin.

In case of crystal violet assay, 150 µl of crystal violet suspension (0.5% wt/v) was added to each well, and the plates were incubated in the dark for 30 min with shaking. The samples were thoroughly washed five times with water to remove the unbound dye. Then, the plates were dried for ~30 min in the dark. Afterward, the bound dye was solubilized in 33% acetic acid for 30 min by shaking at room temperature. The absorbance at 550 nm was recorded to determine the mass of the biofilm.

For resazurin assay, 100 µl of resazurin was added to each well from a stock solution 1 mg ml^−1^ to a final concentration of 4 µg ml^−1^. It was incubated for 30 min at 37 °C and fluorescence was measured at emission wavelength of 590 nm with an excitation wavelength of 550 nm. For the inhibition assay, the formulation was preincubated with the bacteria with OD600 = 0.6 for 52 h, and a resazurin assay was subsequently performed as mentioned above.

#### Antibiofilm properties on the ex vivo human samples

Human molars were procured from Buyamag Inc. (Carlsbad, CA, US) and were sterilized with 70% EtOH. A thick layer of agarose was prepared in the wells of a six-well plate, and then a tooth was placed sideways in each well. Three milliliters of *S. mutans* with OD_600_ = 0.6 was spread on the teeth, and the samples were allowed to rest for 48 h to create the biofilm. The teeth were then transferred to a clean six-well plate and treated for 4 h (3 ml, concentration: 5.7 mM diluted in broth). The teeth were then transferred into centrifuge tubes containing 3 ml of fresh broth and gently and briefly sonicated in a water bath to suspend all the cultivable bacteria in the broth. This media was serially diluted (10^4^, 10^5^, 10^6^, and 10^7^), and 100 μl of each dilution was spread on a BHI agar plate, and the colonies were counted after 24 h.

#### SEM of the biofilm on teeth

The procedure as described above was utilized for growing and treating the biofilms on the teeth. The fixation, drying, and imaging procedures were similar to those described in section for “Scanning electron microscopy with planktonic bacteria”.

### Evaluation of In Vivo Antibiofilm Efficacy

#### In vivo infection of the animals

All animal experiments were carried out according to the ethical guidelines outlined by the Illinois Institutional Animal Care and Use Committee at University of Illinois Urbana-Champaign and were approved by the board (protocol number: 17103). Female Sprague Dawley rats (5-weeks old) were purchased from Charles River Laboratories (Chicago, IL). All the rats were prescreened for any potential indigenous *S. mutans* biofilm with a Saliva Check Mutans Kit (GC America Inc, Alsip, IL). SalivaBio infant swab devices (Salimetrics, LLC, Carlsbad, CA, US) were used for sample collection. The rats (*n* = 3) were randomly placed into three treatment groups: (1) water, (2) CHX, and (3) CHX PR_4_^+^ polymer NPs. The teeth were topically inoculated with 100 μl of *S. mutans* in the mid-log stage of growth using a swab on the incisors on six successive days. A resting period of 7 days was given to allow the infection to set in. At this stage, the *S. mutans* infection was confirmed with the Saliva check kit. The animals were treated daily with 100 μl of formulations by using swabs dipped in the formulation. The treatment concentration was 1.4 mM for 11 days and then 2.3 mM for the last 2 days with a short exposure time of 1 min, mimicking clinical conditions. Before the animals were sacrificed by asphyxiation, they were tested for *S. mutans* infection with the kit. The major organs, teeth, and gums were fixed in formalin and submitted for histopathological examination.

The extracted teeth were immersed in BHI broth, and the bacteria were sonicated off the teeth. The cultivable bacteria were then grown on MSB agar plates those were specific for *S. mutans* growth to avoid contamination by other microbiota present in the oral environment. The number of bacteria was obtained by a colony counting assay to estimate the initial number of *S. mutans*.

#### Histopathological evaluations

Formalin-fixed specimens, including samples of the major organs and gingiva, were dehydrated and embedded in paraffin using a vacuum infiltration tissue processor (Tissue-Tek VIP 6, Sakura Seiki Co, Nagano, Japan). The paraffin-embedded specimens were then sectioned at 3 µm intervals, using a microtome, mounted onto glass slides, dewaxed, and stained with hematoxylin-eosin (H&E) using an automatic slide stainer (Tissue-Tek Prisma A1D, Sakura Finetek Co, Torrance, CA). The staining was performed in hematoxylin solution for 3 min followed by counterstaining in eosin for 1 min. The morphological changes were recorded, scored and verified by a veterinary pathologist at magnifications 20–400×.

#### 16S rRNA Gene Amplicon Sequencing

Dental microbial swabs were collected using sterile Q-tips and stored at 4 °C until further processing. DNA was extracted using a ZymoBIOMICS DNA miniprep kit (Zymo Research), following the procedure provided by the manufacturer. The extracted DNA was sequenced at the Roy J. Carver Biotechnology Center at the University of Illinois, Urbana-Champaign, using primers targeting the V3 and V4 regions of the 16S rRNA gene. Briefly, the concentrations of extracted DNA were measured on a Qubit (Life Technologies) using the High-Sensitivity DNA Kit and subsequently diluted to 2 ng μl^−1^. For PCR amplification, a mastermix for amplification was prepared using the Roche High Fidelity Fast Start Kit according to the Fluidigm protocols. For each sample, the following reagents were mixed: 0.5 μl of 10× Fast Start Reaction Buffer (without MgCl_2_), 0.9 μl of 25 mM MgCl_2_, 0.25 μl of DMSO, 0.1 μl of 10 mM PCR-grade nucleotide mix, 0.05 μl of 5 U μl^−1^ Fast Start High Fidelity Enzyme Blend, and 1.0 μl of primer mix for V3 and V4. The primer sequences were as follows: V3-F 5′-CCTACGGGNGGCWGCAG-3′, V4-R 5′-GACTACHVGGGTATCTAATCC-3′. To each well, 1.2 μl of DNA sample and 1 μl of Fluidigm Illumina linkers with unique barcodes were added for library prep. A single-step PCR protocol was performed for the amplification of DNA sequences and the addition of barcodes. The PCR product was quantified on a Qubit fluorimeter and stored at −20 °C until use. All samples were run on a Fragment Analyzer (Advanced Analytics), and the amplicon regions and expected sizes were confirmed. The samples were then pooled in equal amounts according to product concentration. The pooled products were size selected on 2% agarose E-gel (Life Technologies) and extracted from the isolated gel slice with a Qiagen gel extraction kit (Qiagen), using a Qiacube robot. The cleaned, size-selected products were evaluated on an Agilent Bioanalyzer to confirm the appropriate profile and determine their average size.

The library was quantified by qPCR and sequenced using a MiSeq Nano flowcell for 251 cycles from each end of the fragments using a MiSeq 500-cycle sequencing kit version 2. FASTQ files were generated and demultiplexed with bcl2fastq v2.20 Conversion Software (Illumina).

#### Processing of sequence data

Demultiplexed sequence data were submitted to the dada2 high-throughput Nextflow^94^ pipeline on the Biocluster at the Carl R. Woese Institute for Genomic Biology at UIUC. R version 3.5.0 (ref. ^95^) was used in the HPC analysis steps, and the primary data processing steps were handled by dada2 v1.10 (ref. ^96^) and DECIPHER v2.10 (ref. ^97^). The ASV count table, rank assignments, phylogenetic tree, and experimental data were analyzed using R Studio, phyloseq^98^ and vegan^99^ using an RMarkdown script. The initial microbial composition plots were generated, and that was followed by alpha-diversity analysis using Shannon index metrics. Beta diversity analysis was performed using weighted UniFrac distances. Principal coordinate analysis was performed using QIIME. This analysis is a multivariate statistical technique for finding the most important dimensions along which samples vary.

### Statistics and reproducibility

The results were expressed as the mean ± the standard deviation, and proper statistical analysis was performed on GraphPad Prism 6.0 software, as noted for the individual experiments.

The following files are available free of charge: Figs. S1 – S30.

### Reporting summary

Further information on research design is available in the Nature Research Reporting Summary linked to this article.

## Supplementary information

Supplementary information

Description of Supplementary Files

Supplementary Data 1

Reporting Summary

## Data Availability

The RNA-sequencing data are openly available in Zenodo^100^. Source data underlying plots shown in figures are provided in Supplementary Data 1. All other data are available upon reasonable request.
