# Supplementary material for: Function-adaptive clustered nanoparticles reverse Streptococcus mutans dental biofilm and maintain microbiota balance
Source: Commun Biol. 2021 Jul 15;4:846. doi: 10.1038/s42003-021-02372-y (PMC8282845; doi:10.1038/s42003-021-02372-y)
Supplement: Supplementary file 1 — Supplementary information [file 42003_2021_2372_MOESM1_ESM.pdf]

Supplementary Information

**Function-adaptive Clustered Nanoparticles Reverse Streptococcus Mutans Dental Biofilm and Maintain Microbiota Balance**

*Fatemeh Ostadhossein* [1], *Parikshit Moitra* [2], *Esra Altun* [1,†], *Debapriya Dutta* [1,†], *Dinabandhu Sar* [1,†], *Indu Tripathi* [1,†], *Shih-Hsuan Hsiao* [3], *Valeriya Kravchuk* [1], *Shuming Nie* [4], *Dipanjan Pan* [*1,2,5,6]

[1] Departments of Bioengineering, Beckman Institute, University of Illinois at Urbana-Champaign, Mills Breast Cancer Institute, and Carle Foundation Hospital, Urbana, Illinois 61801, USA.

[2] Department of Pediatrics, Center for Blood Oxygen Transport and Hemostasis, Health Sciences Facility III, University of Maryland Baltimore School of Medicine, 670 W Baltimore St., Baltimore, Maryland, 21201, USA

[3] Veterinary Diagnostic Laboratory, University of Illinois at Urbana-Champaign, Illinois, USA

[4] Departments of Bioengineering, Carle Illinois College of Medicine, Beckman Institute, Department of Chemistry, Department of Materials Science and Engineering, University of Illinois at Urbana-Champaign, Urbana, Illinois 61801, United States

[5] Department of Diagnostic Radiology and Nuclear Medicine, Health Sciences Facility III, University of Maryland Baltimore, Baltimore, Maryland, 21201, United States

[6] Department of Chemical, Biochemical and Environmental Engineering, University of Maryland Baltimore County, Interdisciplinary Health Sciences Facility, 1000 Hilltop Circle Baltimore, Maryland, 21250, United States

ǂ,†The authors contributed equally to this work.

*To whom correspondence should be addressed: E-mail: dipanjan@illinois.edu, dipanjan@som.umaryland.edu

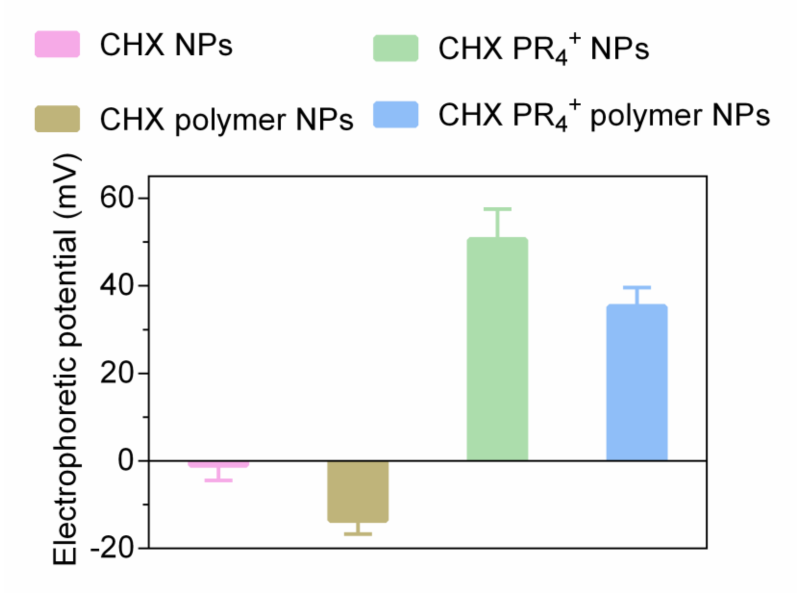


**Figure S1.** Electrophoretic ζ-potential of various nanoparticles used in this study. The values are directly read as mean ± SD from the device.

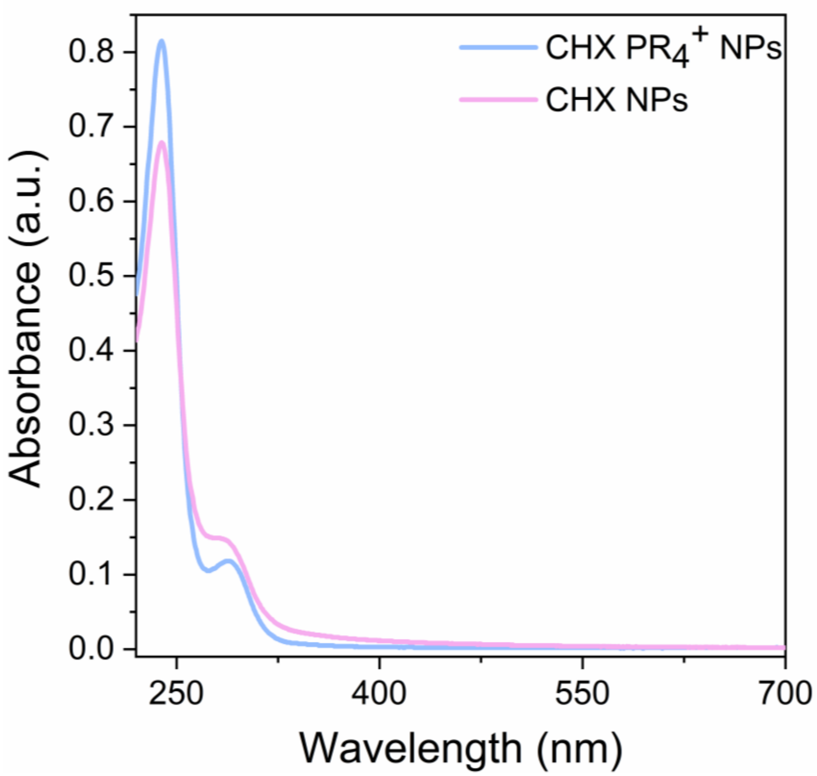


**Figure S2.** UV-Vis absorbance of CDot core for CHX and CHX $PR_4^+$ NPs.

The absorption spectra of CHX NPs is lacking any bandgap transition that is due to the amorphous nature of the carbon substrate. The characteristic peak of CHX NPs around 289 nm was observed and was attributed to the n−π* transition of the carbonyl group and π–π* transition of the conjugated C=C band. This peak got slightly red-shifted to 292 nm for the CHX $PR_4^+$ NPs.

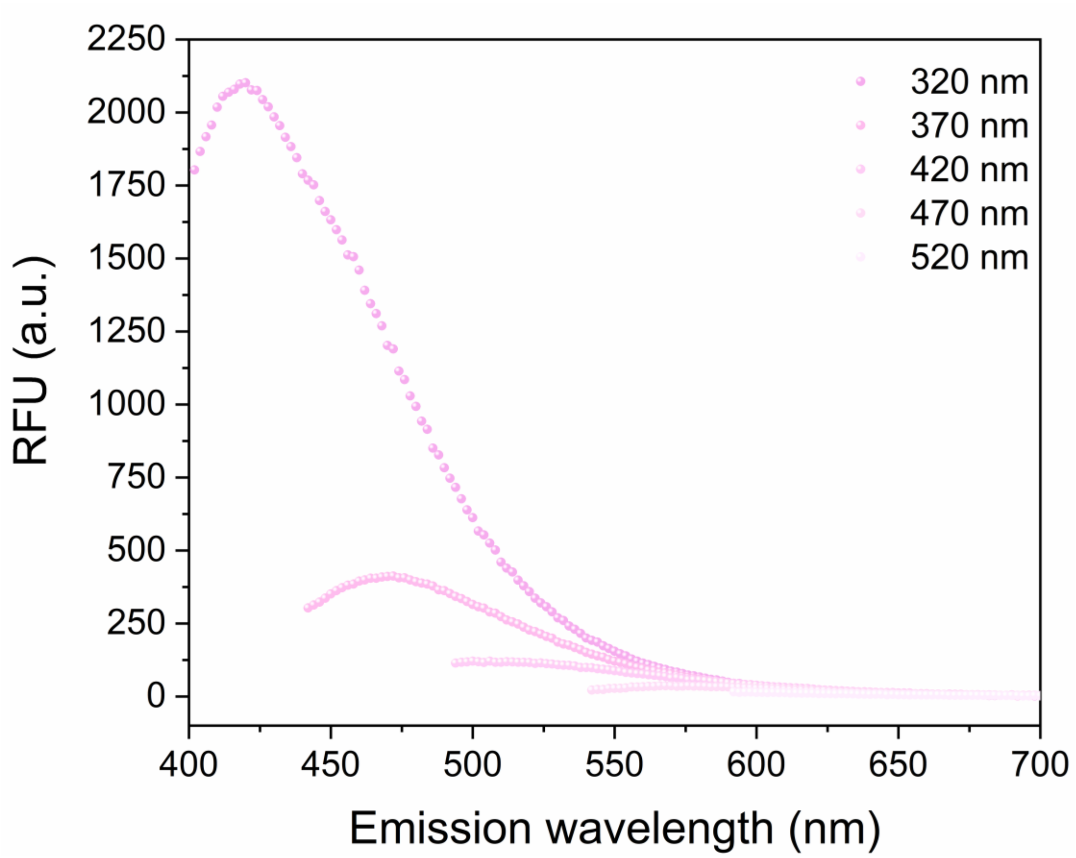


**Figure S3.** Excitation dependent emission fluorescence of CHX NPs at different excitation wavelengths.

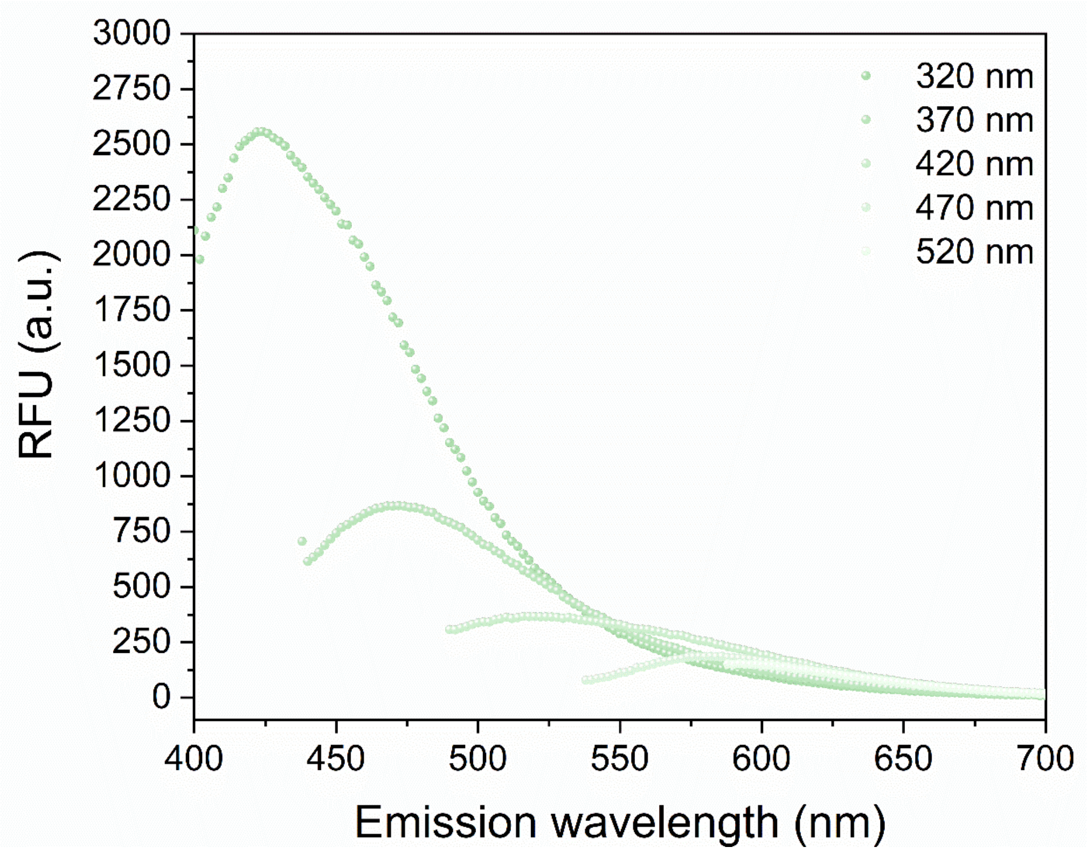


**Figure S4.** Excitation dependent emission fluorescence of CHX $PR_4^+$ NPs at different excitation wavelengths.

The fluorescence spectra of CHX NPs and CHX $PR_4^+$ NPs displayed a typical excitation dependent photoluminescence of CDots in the blue-green region. This behavior can be associated with the difference in the response of the multiple emission centers of CDots, either on the surface (*e.g.* surface fluorophores) or their core (*e.g.* extended conjugating in the graphitic core).

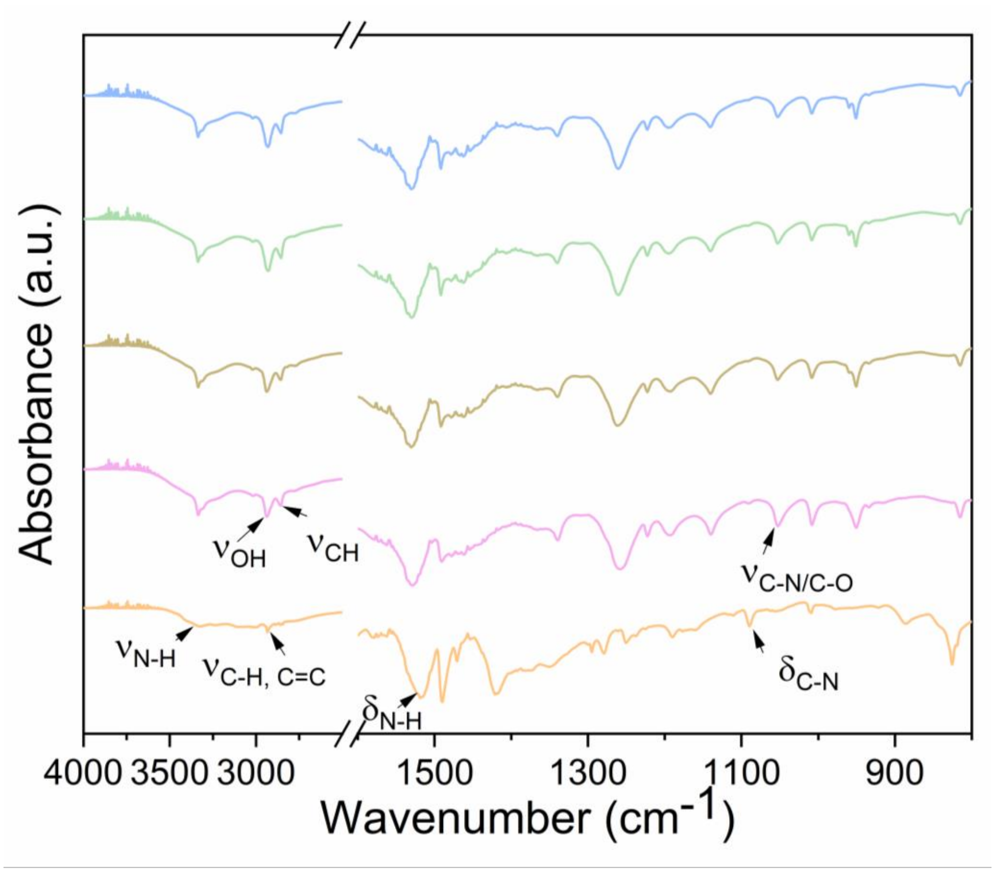


**Figure S5.** Fourier transform infrared (FTIR) spectra of CHX, CHX NPs, CHX polymer NPs, CHX $PR_4^+$ NPs, CHX $PR_4^+$ polymer NPs respectively from bottom to top.

In the CHX profile, several characteristic peaks were identified: the peak at 1090, 1518, 1644 and 2934 $cm^{-1}$ can be attributed to $\delta_{C-N}$ aliphatic, $\delta_{N-H}$, aromatic C=C, and C-H, respectively. On the other hand, the broad peak between 3283-3395 $cm^{-1}$ can be assigned to N-H. After the carbonization of CHX to CDots (*i.e.* CHX NPs), there is no overlap between the two spectra. In the spectrum of CHX NPs several regions can be identified. The region between 3208-3363 $cm^{-1}$ can be due to -OH stretch in alcohols and N-H stretch. The strong major absorbance centered at 2938 $cm^{-1}$, 2856 $cm^{-1}$ may be due to OH stretch in COOH and C-H stretch in alkane respectively. The fingerprint region can be split into several areas: absorbances seen at 1053, 1400-1450, 1524, and 1630-1690 $cm^{-1}$ can be related to C-N/ C-O, OH bend in COOH, C=C, C=N/ amide, respectively.

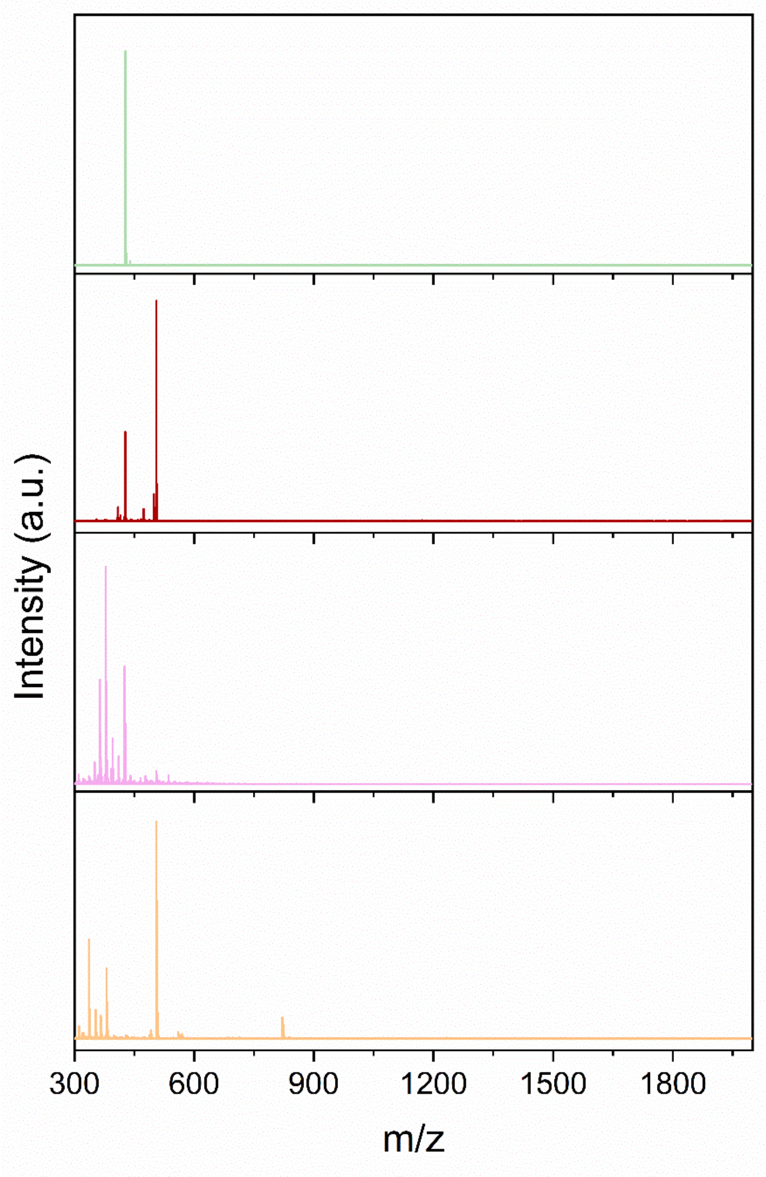


**Figure S6.** Chemical composition characterizations of the NPs by mass spectroscopy analysis of CHX, CHX NPs, $PR_4^+$, CHX $PR_4^+$ NPs (from bottom to top) where the lack of CHX characteristic m/z signal is perceivable after carbonization. For CHX: 336.1, 380.7, 428.7, 505.3 (strong), 559.6, 821.4; For CHX NPs: 335.2 (suppressed), 350.3, 363.2, 378.2 (strong), 425.5, 505.4, 582.5; For $PR_4^+$: 427.1, 473.1, 505.1, For CHX $PR_4^+$: 355 (strong), 393.6, 451.1, 505.1.

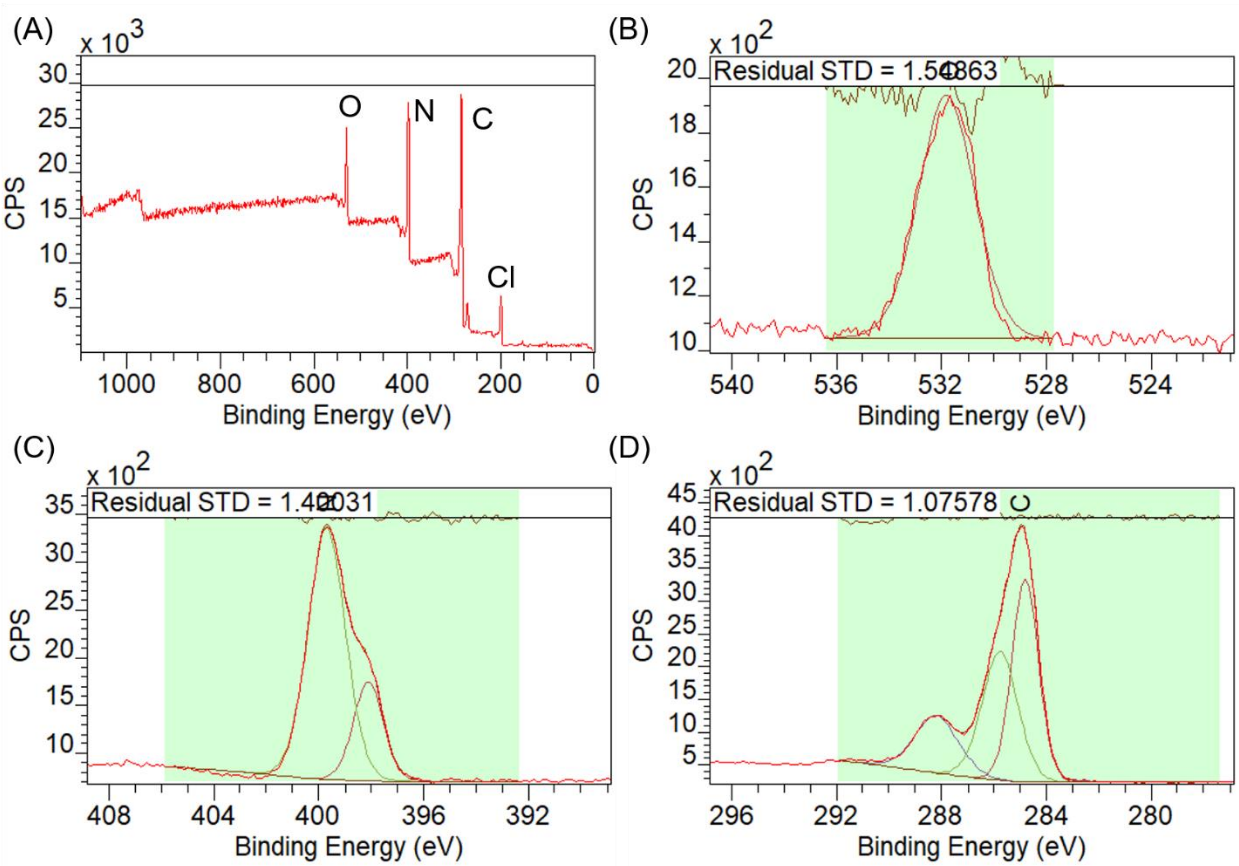


**Figure S7.** XPS analysis for CHX (A) survey mode, and decomposed peaks for (B) O1s, (C) N1S, and (D) C1s.

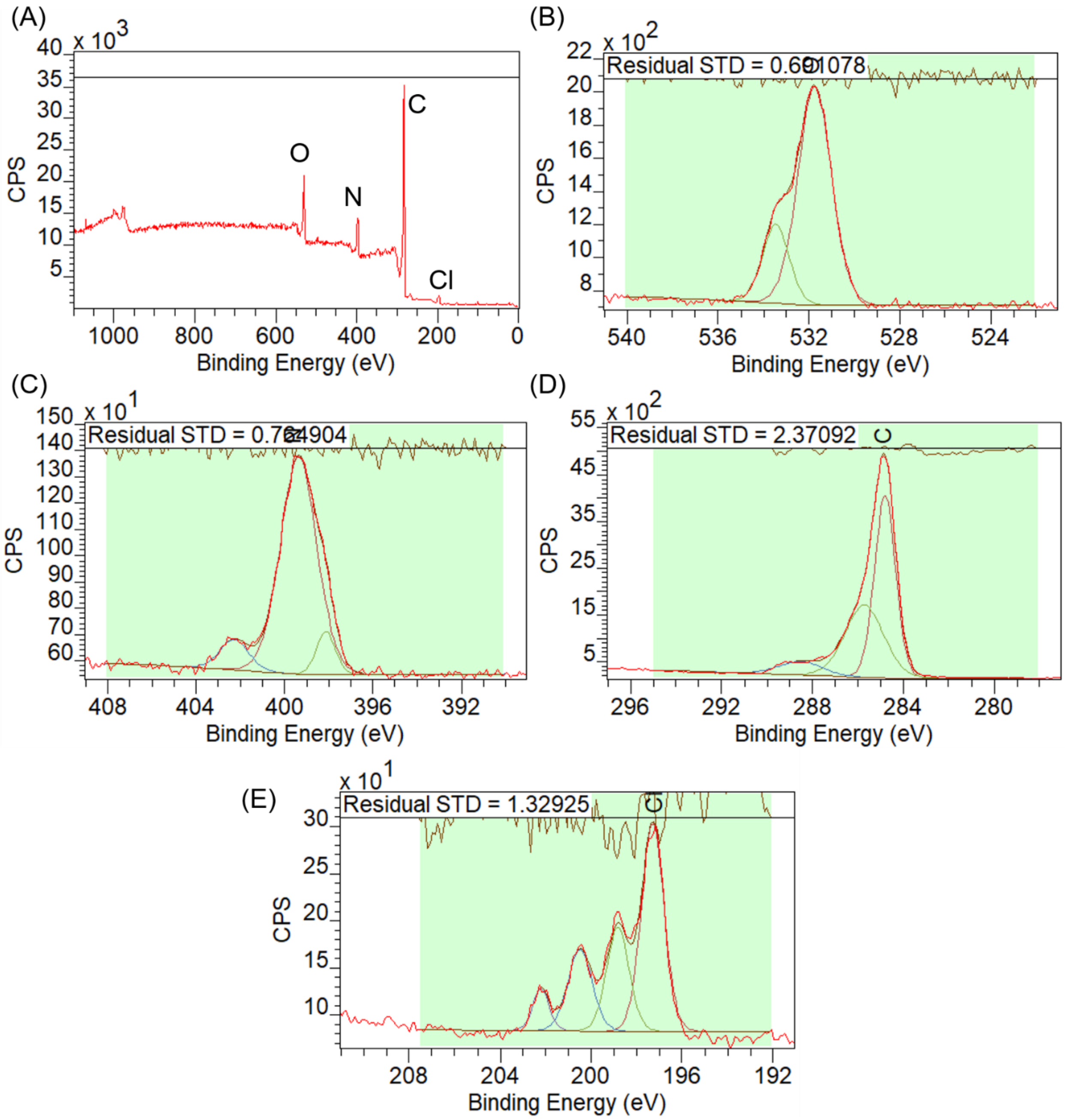


**Figure S8.** XPS analysis for CHX NPs (A) survey mode, and decomposed peaks for (B) O1s, (C) N1S, (D) C1s, and (E) Cl2p.

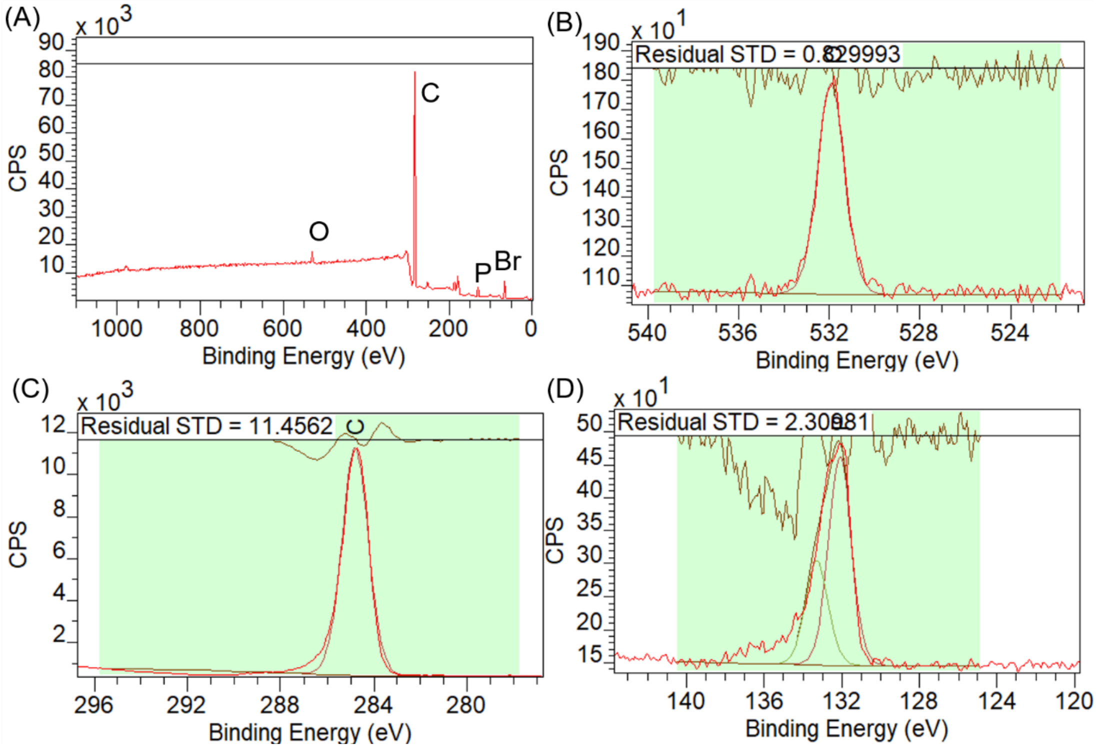


**Figure S9.** XPS analysis for $PR_4^+$ compound (A) survey mode, and decomposed peaks for (B) O1s, (C) C1s, and (D) Br3d.

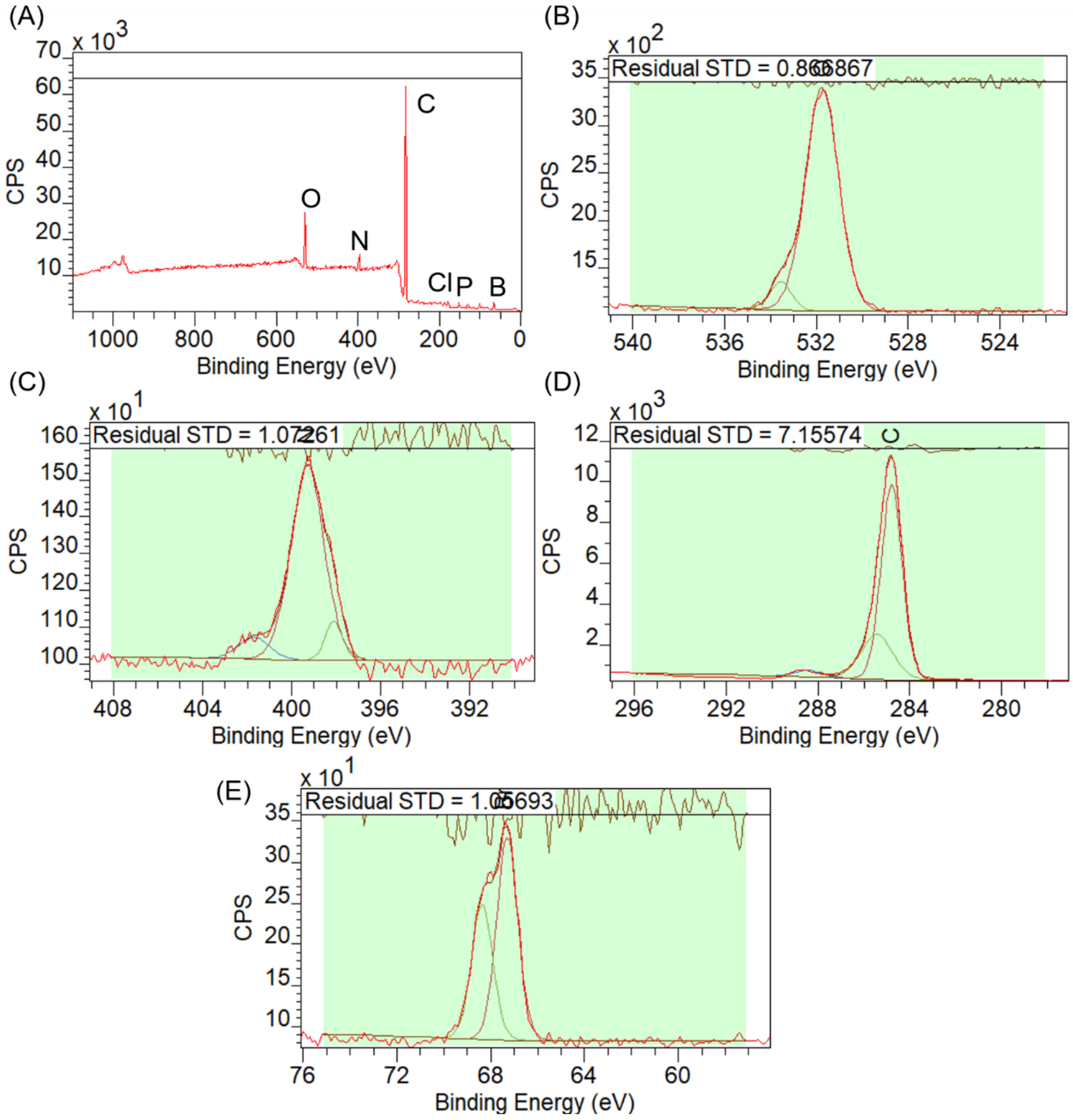


**Figure S10.** XPS analysis for $PR_4^+$ compound (A) survey mode, and decomposed peaks for (B) O1s, (C) N1s, (D) C1s, and (E) Br3d.

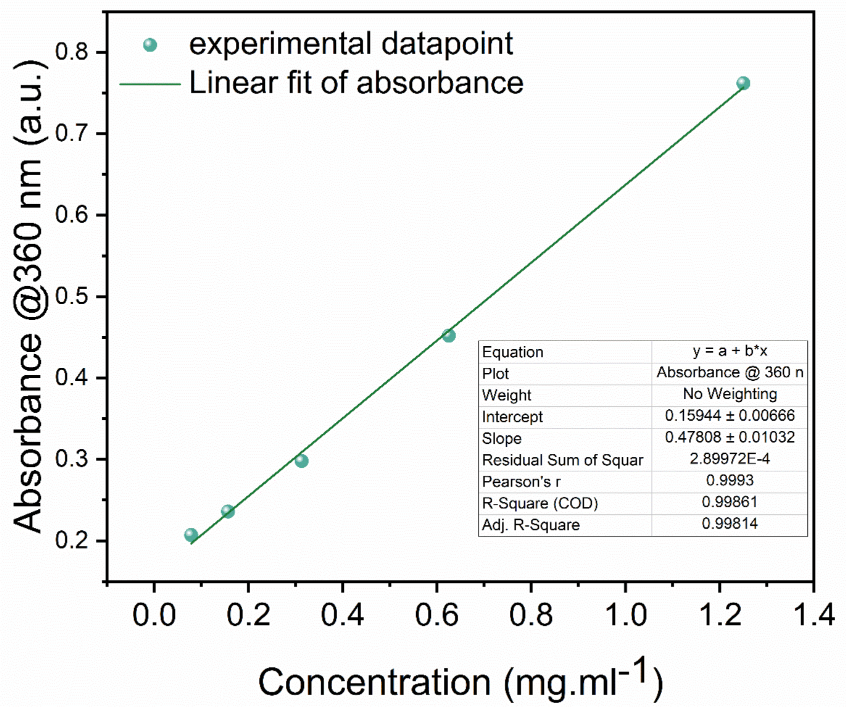


**Figure S11.** The calibration curve used for the calculation of the percent released nanoparticles. The absorbance at 360 nm was recorded over time for the dialysate. The absorbance of 2.5 mg.ml-1 of NPs was 1.5 as the 100% release criteria.

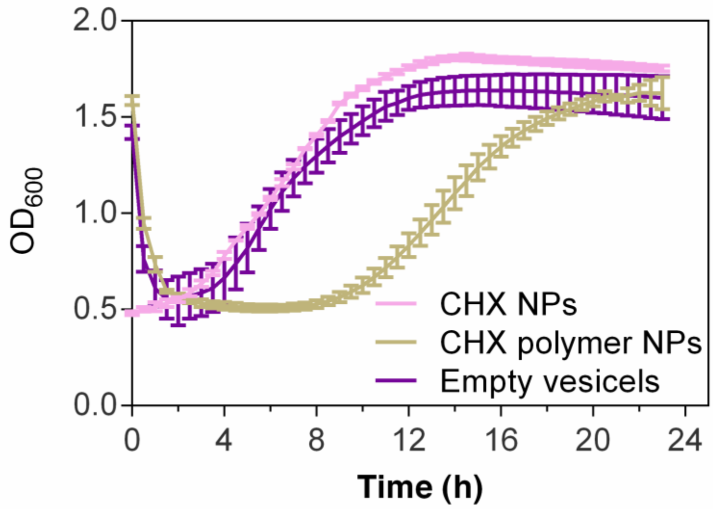


**Figure S12.** Time- kill assay for CHX NPs, CHX polymer NPs and empty vesicle NPs where the growth of bacteria was observed over time. $OD_{600}$ is the absorbance at 600 nm in arbitrary unit (a.u.).

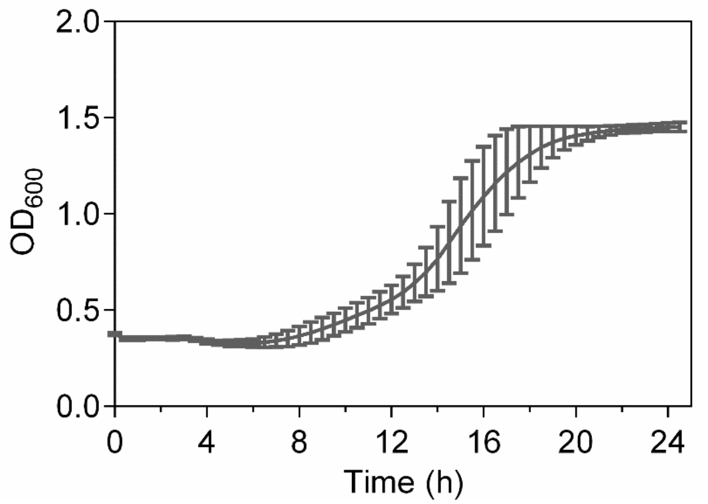


**Figure S13.** Bacterial growth in water with time. $OD_{600}$ is the absorbance at 600 nm in arbitrary unit (a.u.).

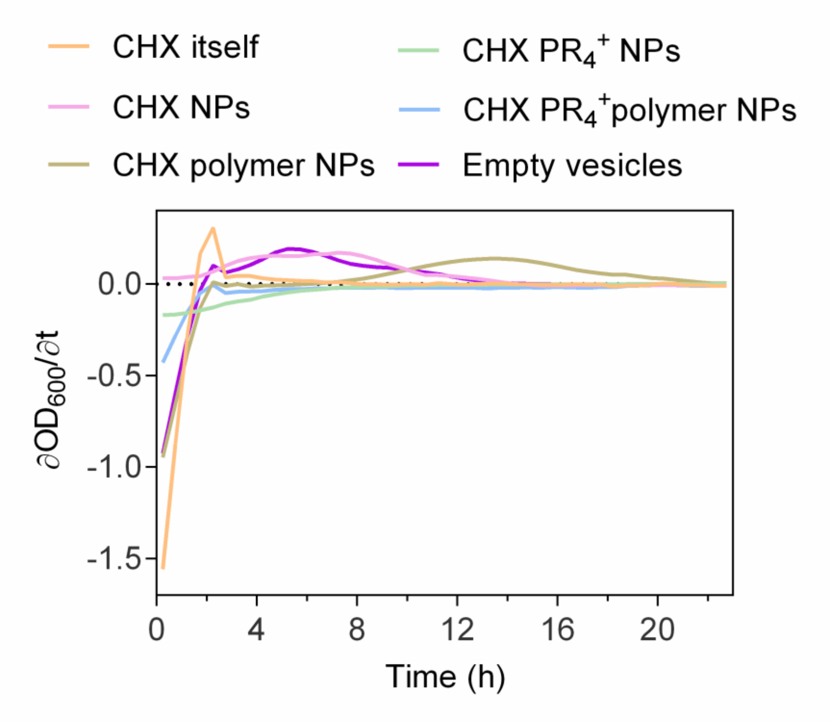


**Figure S14.** The first order derivative of $OD_{600}$ vs. time. $OD_{600}$ is the absorbance at 600 nm in arbitrary unit (a.u.). The change in slope is more prominently shown in the first order derivate plot where the negative value of the slope is preserved all along for CHX NPs and CHX polymer NPs while the trend is more variable for the rest of the samples. The first order derivative plot would allow exclusion of turbidity and scattering from the NPs.

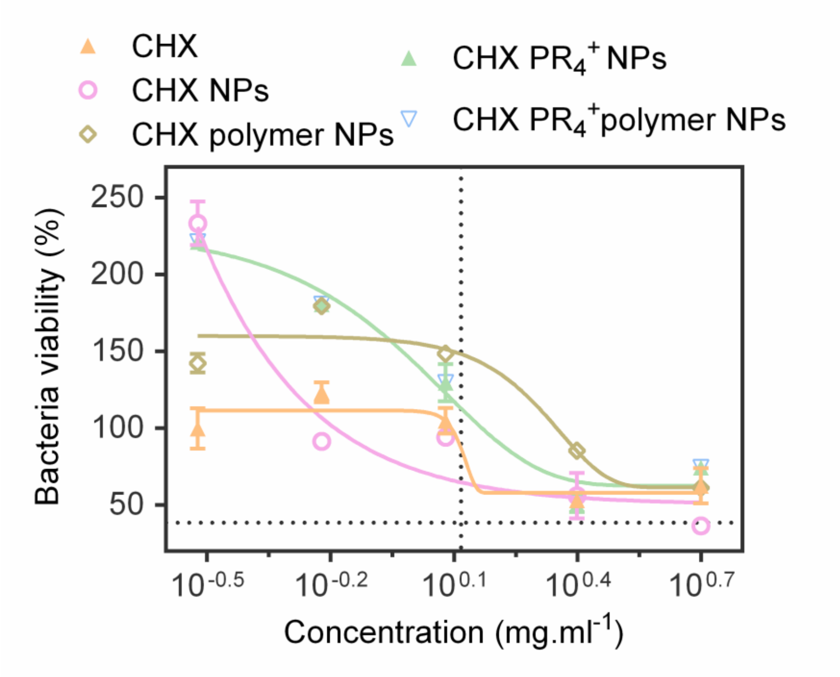


**Figure S15.** MIC value determination. $\lambda_{600}$ was read and normalized for various concentrations and a viability curve vs. log(concentration) was established to calculate MIC at 20 h. The value was determined to be 2.026 mg.ml$^{-1}$ for both CHX NPs ($R^2$=0.999) and CHX polymer NPs ($R^2$=0.975) according to Lambert and Pearson's analysis method (1). This translates to 5.70 mM of therapeutic CDots given the molecular mass of 355 from mass spec results.

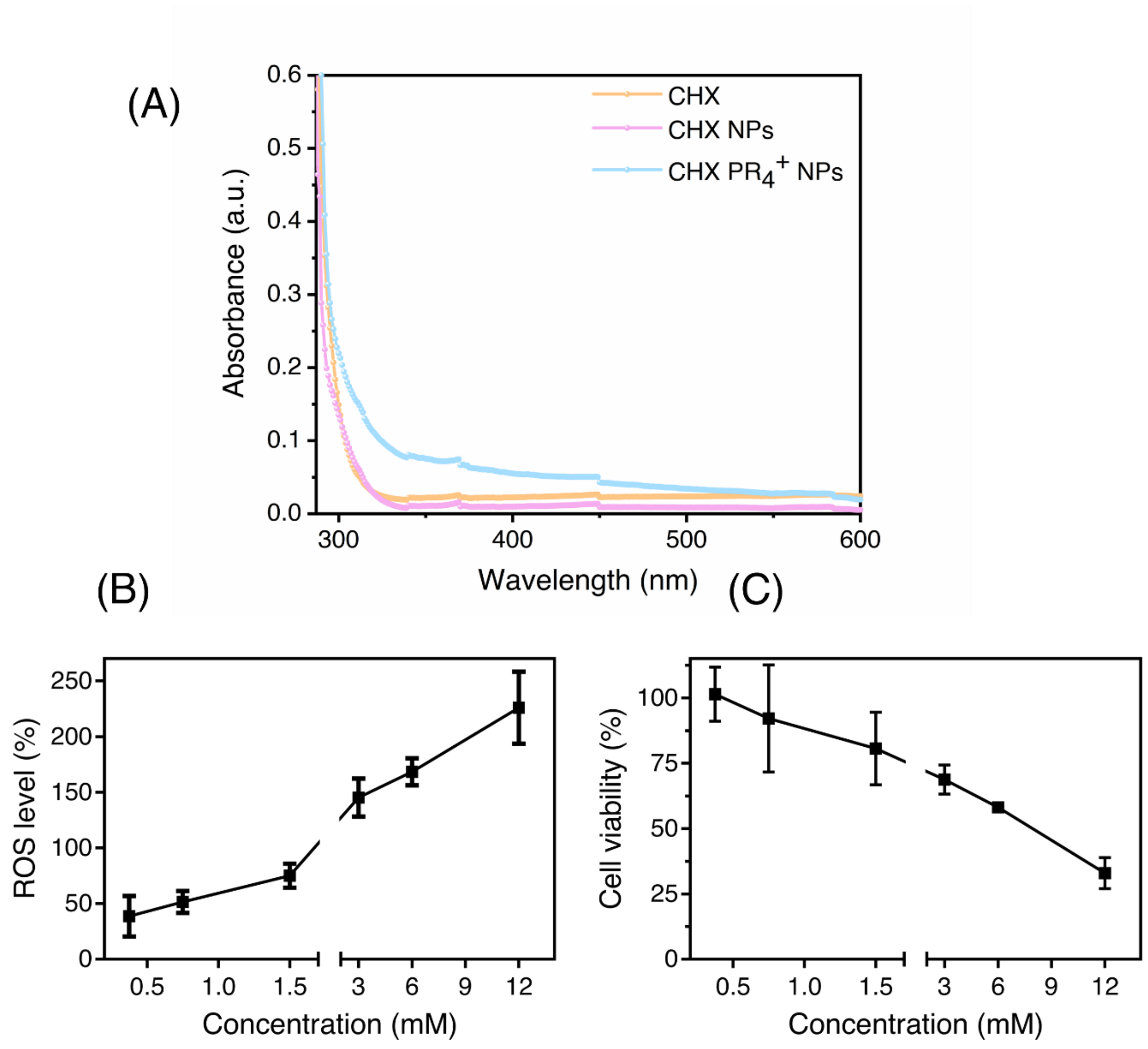


**Figure S16.** The UV Vis spectra for the bandgap calculation, (B) ROS generation in the NIH 3T3 cells and (C) Cell viability of the NIH 3T3 cells.

Either high HOMO or low LUMO level will indicate the increased electron accepting property of the nanoparticles. This can also be a reason behind the increased ROS level in CHX-$PR_4^+$ polymer NP group than that of the positive control (CHX) group.

For CHX and CHX NPs, $\lambda$ = 310 nm

Eg = hc/$\lambda$ = 1240/310 (eV) = 4 eV = 0.147 a.u.

For CHX $PR_4$+ Polymer NPs, $\lambda$ = 400 nm

Eg = hc/$\lambda$ = 1240/400 (eV) = 3.1 eV = 0.114 a.u.

HOMO-LUMO gap is less for CHX $PR_4^+$ Polymer NPs. For this, it will have increased electron accepting property and increased oxidant generating property.

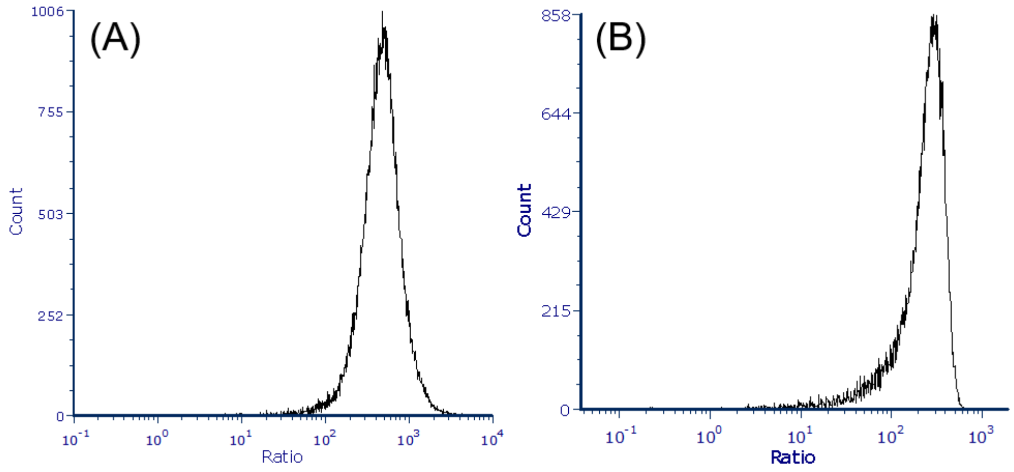


**Figure S17.** The ratiometric parameter distribution in polarization assay. (A) control water treatment (B) CHX $PR_4^+$ polymer NP treatment.

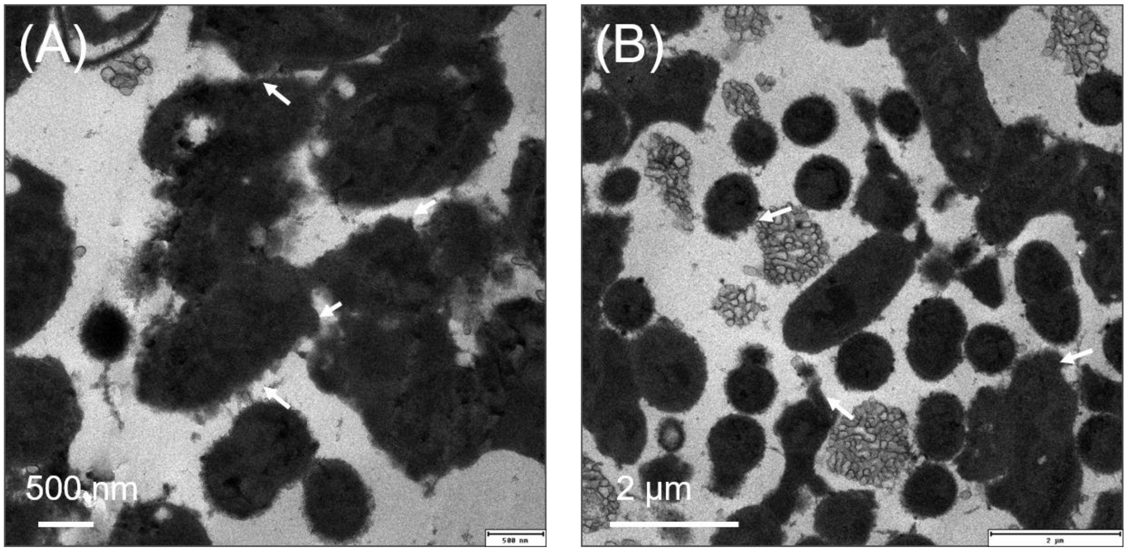


**Figure S18.** The lower magnification TEM images of bacteria treated with CHX $PR_4^+$ polymer NPs.

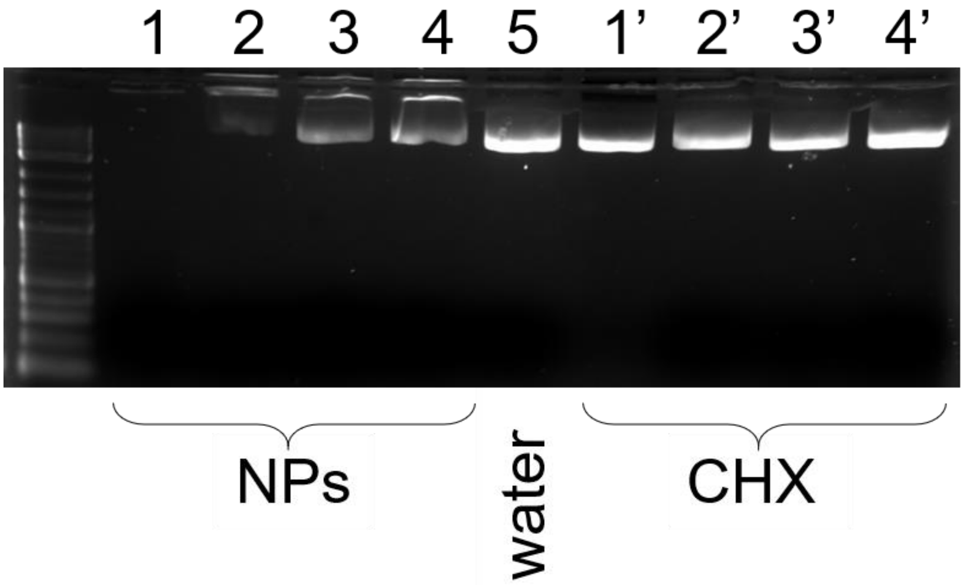


**Figure S19.** pDNA interaction and damage after 1 h incubation with CHX $PR_4^+$ polymer NPs and CHX, lane order is: ladder, 1: 5.7 mM, 2: 1.4 mM, 3: 0.35 mM, 4: 0.09 mM of NPs, 5: water, 1': 5.7 mM, 2': 1.4 mM, 3': 0.35 mM, 4'0.09 mM of CHX. As is shown, the lanes for the CHX $PR_4^+$ polymer NPs are fainter compared to both CHX and water treatment group implying their effective interaction and damage of the pDNA.

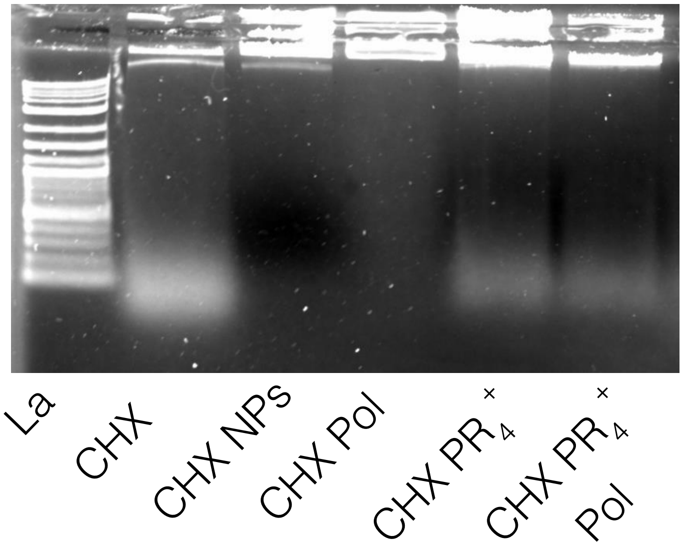


**Figure S20.** Genomic DNA interaction with various formulations after treatment for 4 h. Note that Figure 3E is extracted from this result but some lanes were not shown for keeping the flow of the text.

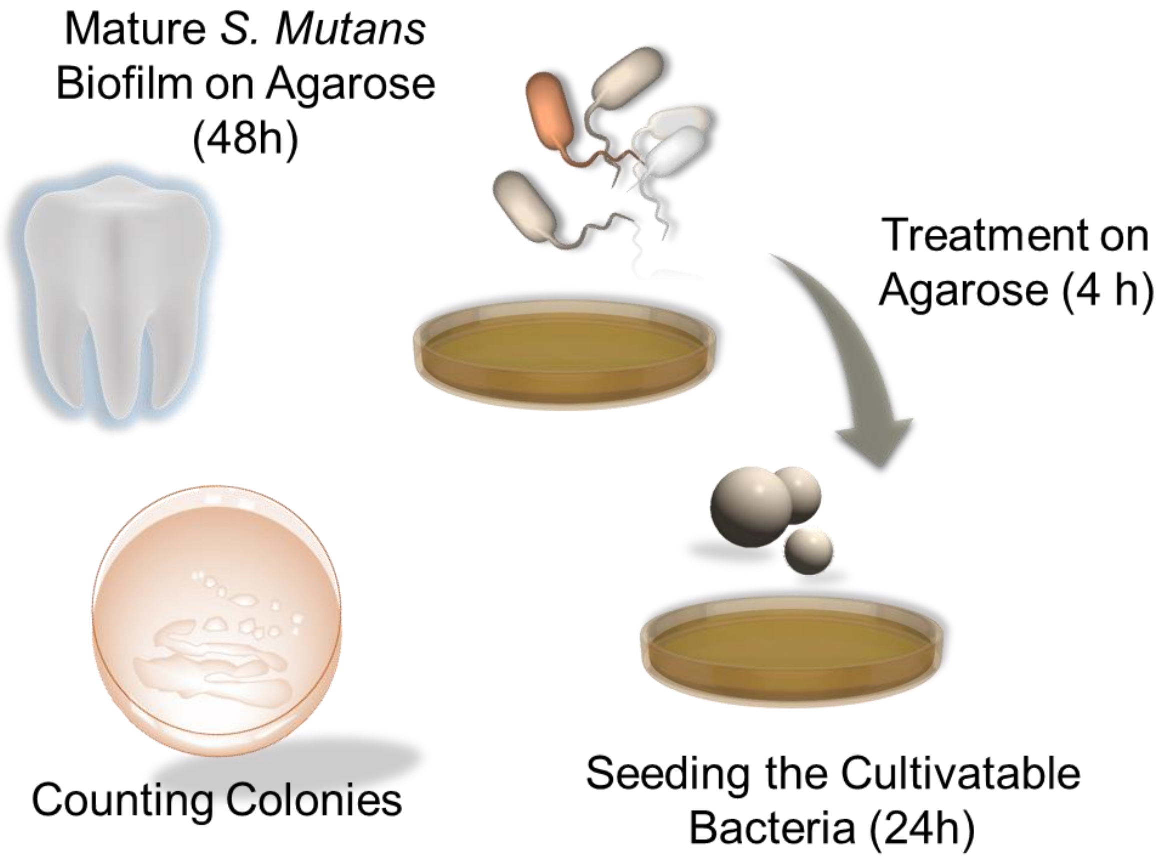


**Figure S21.** The flowchart demonstrating the *ex vivo* experimental setup for the growth of biofilm on human teeth and subsequent treatment. The components of the image were made available free of charge from https://www.somersault1824.com/.

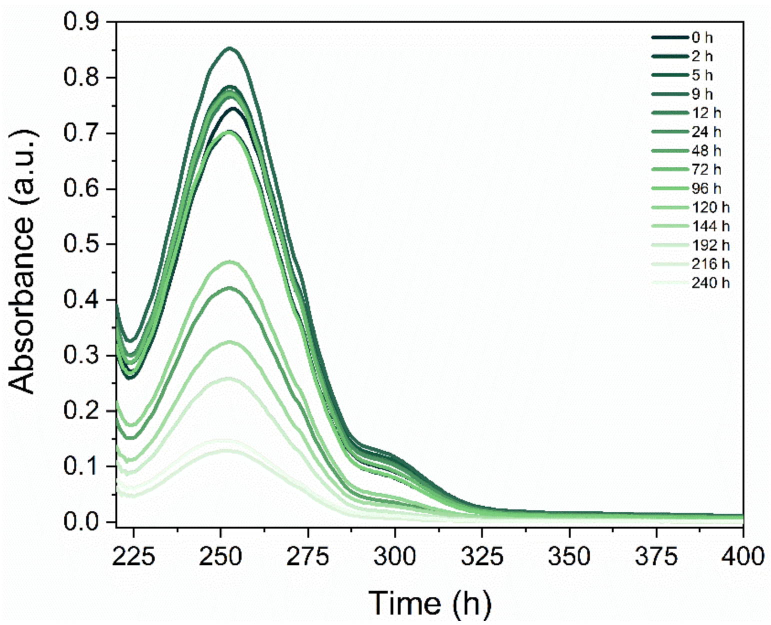


**Figure S22.** The UV-Vis spectra of CHX $PR_4^+$ NPs in the artificial saliva for various time points.

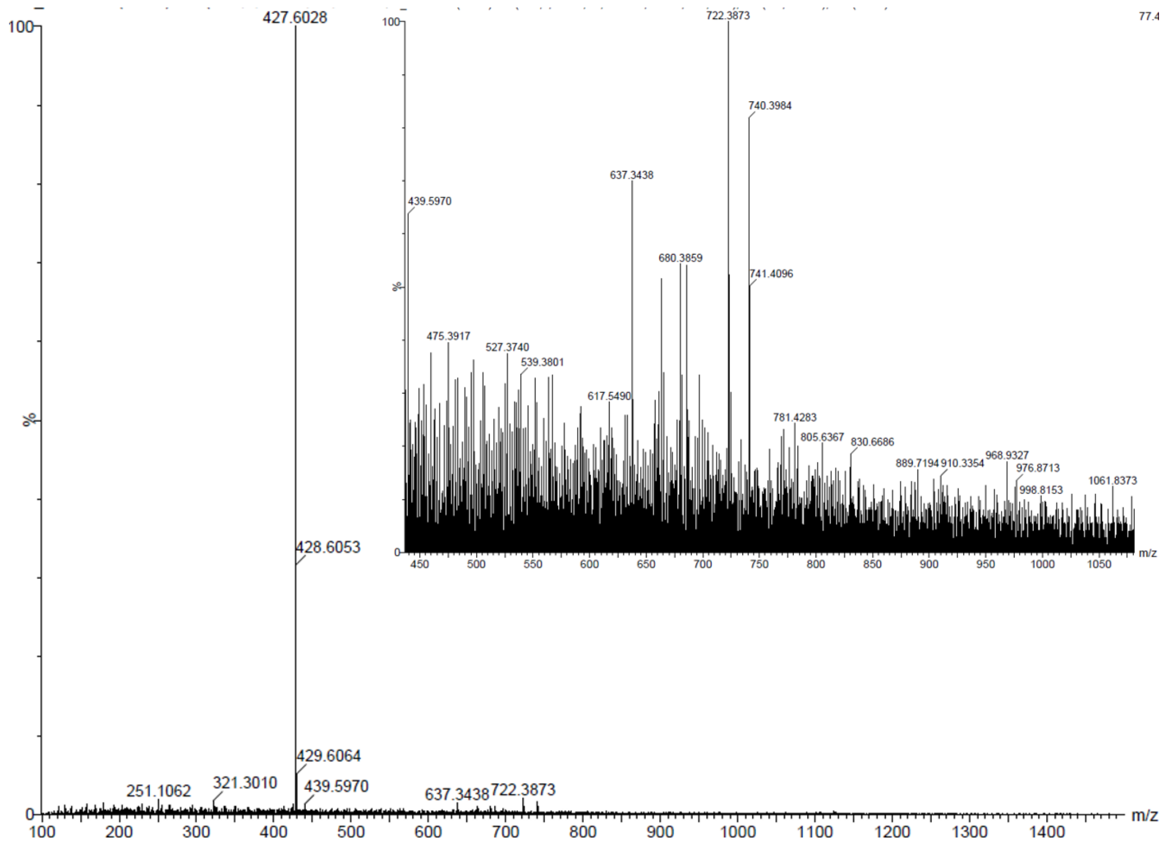


**Figure S23.** ESI mass spectrometry results for the degraded product on day 1.

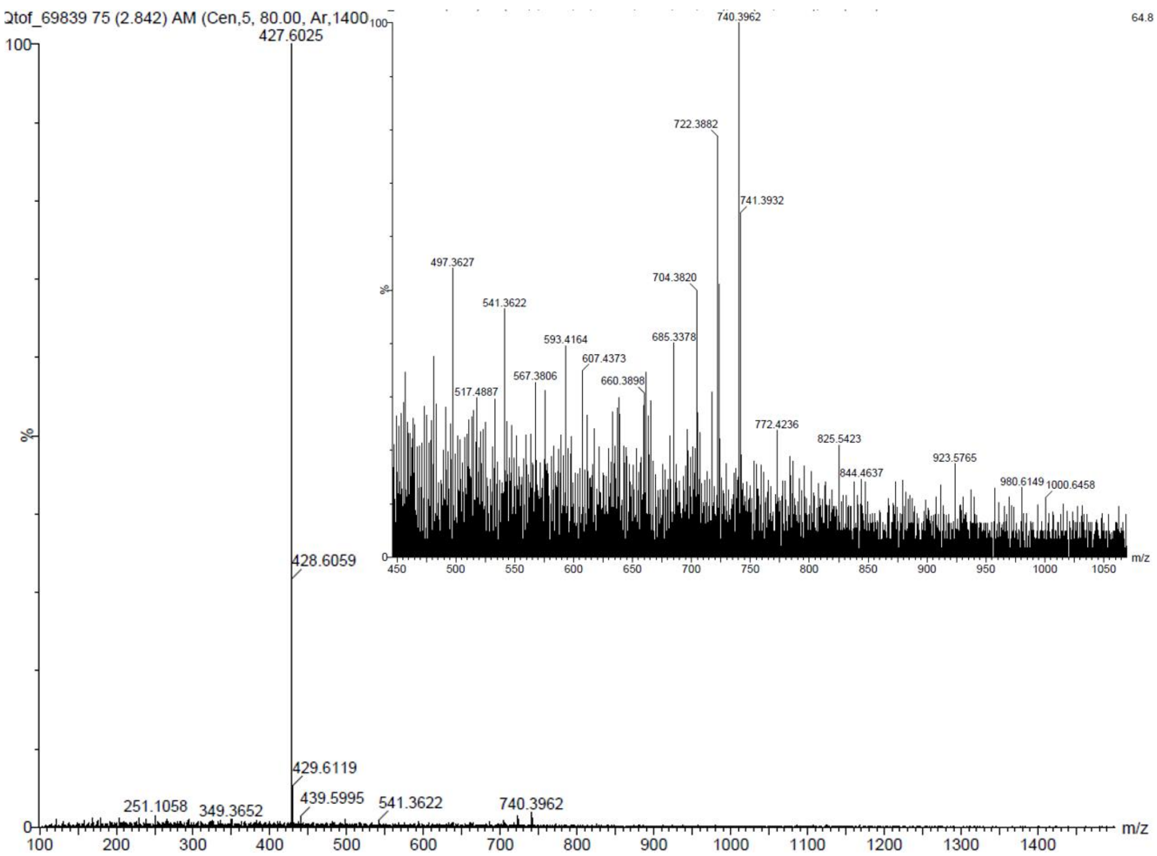


**Figure S24.** ESI mass spectrometry results for the degraded product on day 2.

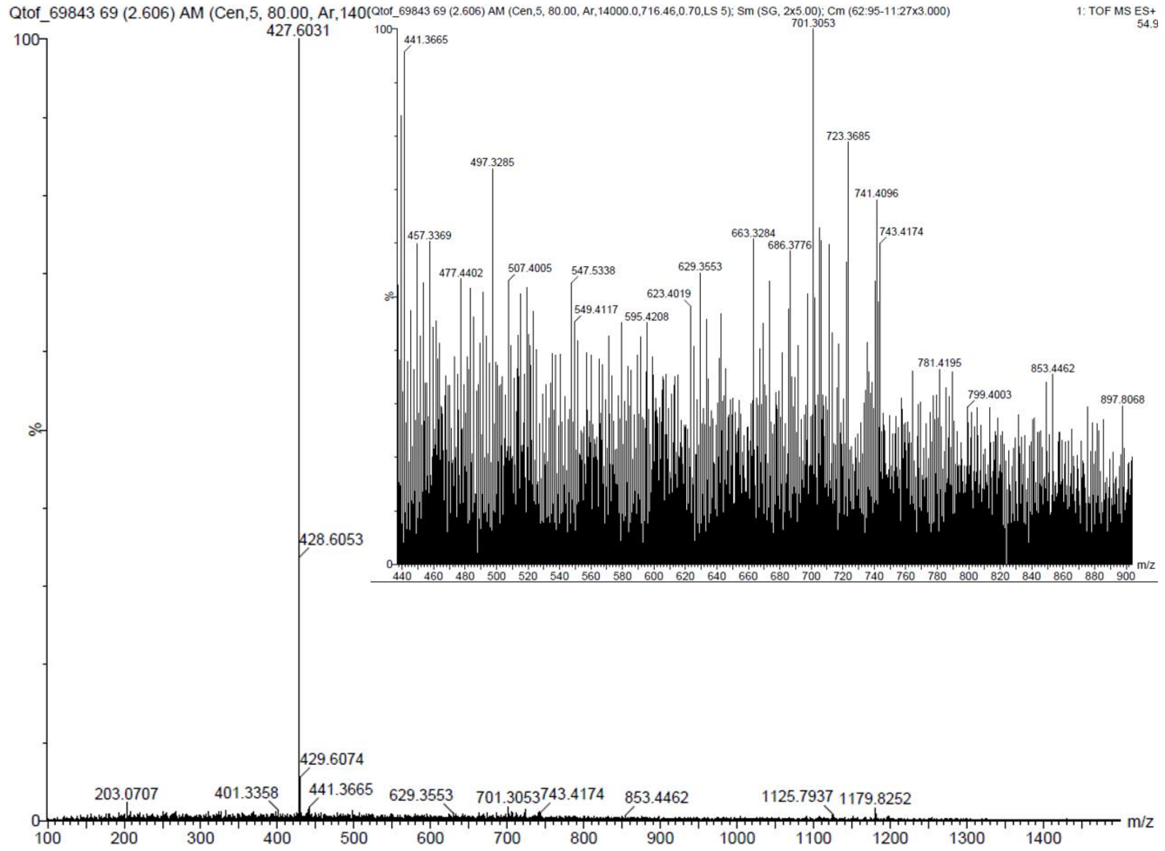


**Figure S25.** ESI mass spectrometry results for the degraded product on day 8.

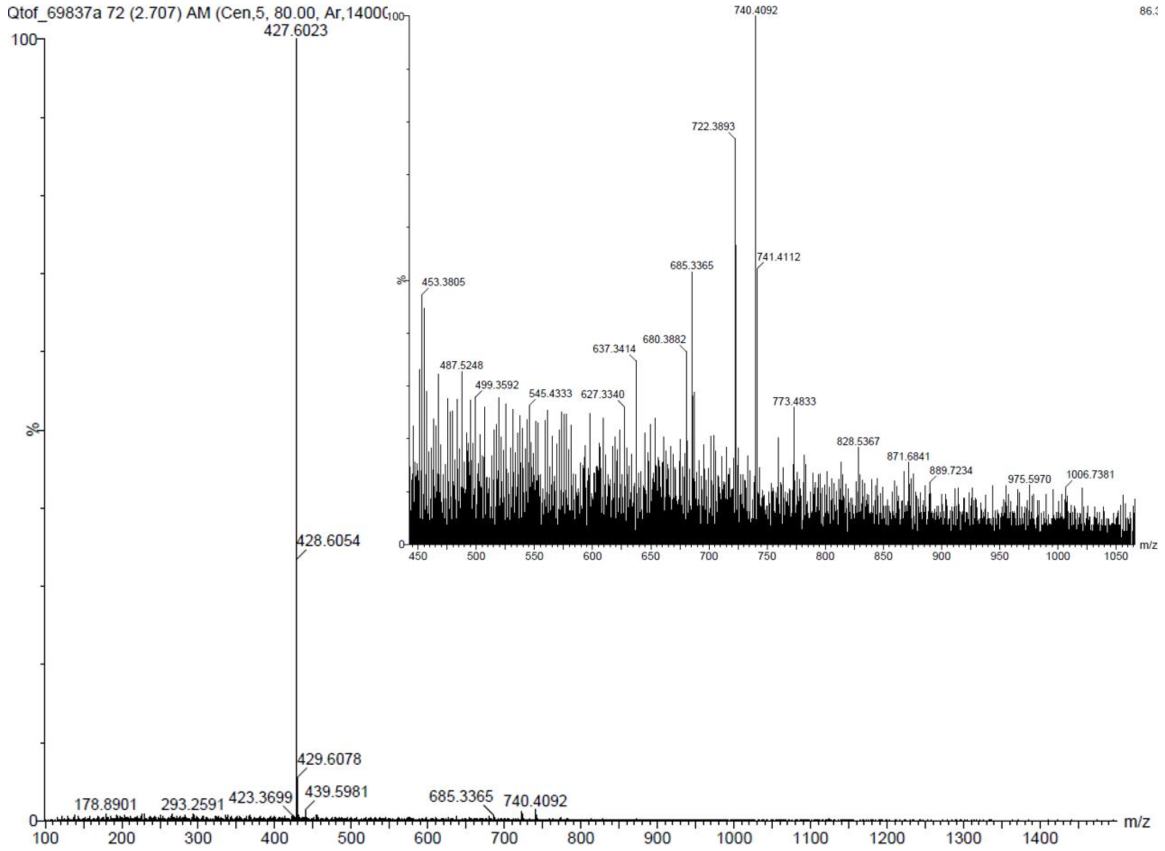


**Figure S26.** ESI mass spectrometry results for the degraded product on day 10.

**The proposed calculations for the mass spectrometry results on different days:**

**0h**

(2 x 127) + 170 +3H$^+$ = 427

(3 x 170) + 127 = 637

(2 x 352) = 704

(2 x 352) + 1H$^+$ = 705

352 + 170 + 200 = 722

(2 x 170) + (2 x 200) = 740

(2 x 170) + (2 x 200) + 1H$^+$ = 741

$(2x200) + 353 + 2H^+$ =755

**1d**

353 + 200 + 127 =680

(3 x 127) + (2 x 200) = 781

**2d**

352 + 127 + $NH_4^+$ = 497

353 + 200 + 127 + $5H^+$ =685

(3 x 200) + 170 + $2H^+$ = 772

**8d**

353 + 200 + 127 + $6H^+$ =686

170 + (2 x 200) + 127 + $4H^+$ = 701

353 + 170 + 200 = 723

(2 x 170) + (2 x 200) + $3H^+$ = 743

**10d**

(3 x 200) + 170 + $3H^+$ = 773

(5 x 127) + 170 +200 + $Na^+$ = 828

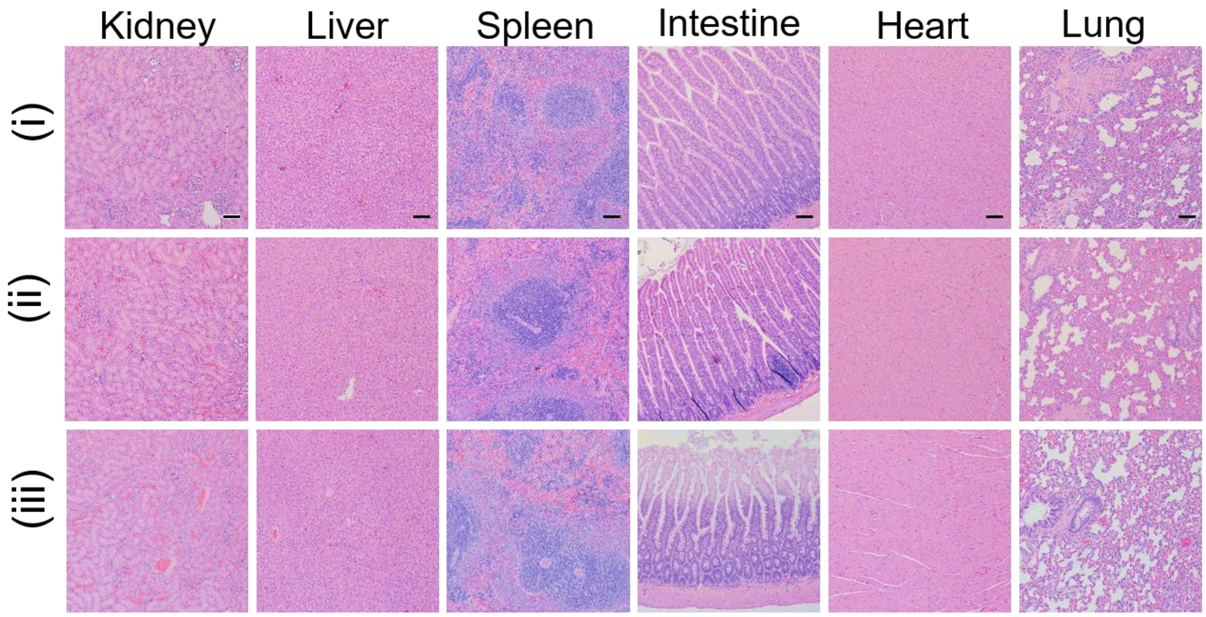


**Figure S27.** H&E histological analyses of the major organs: (i) water, (ii) CHX, (iii) CHX $PR_4^+$ polymer NP treated group. No significant changes were observed in the groups. The scale bar corresponds to 100 μm.

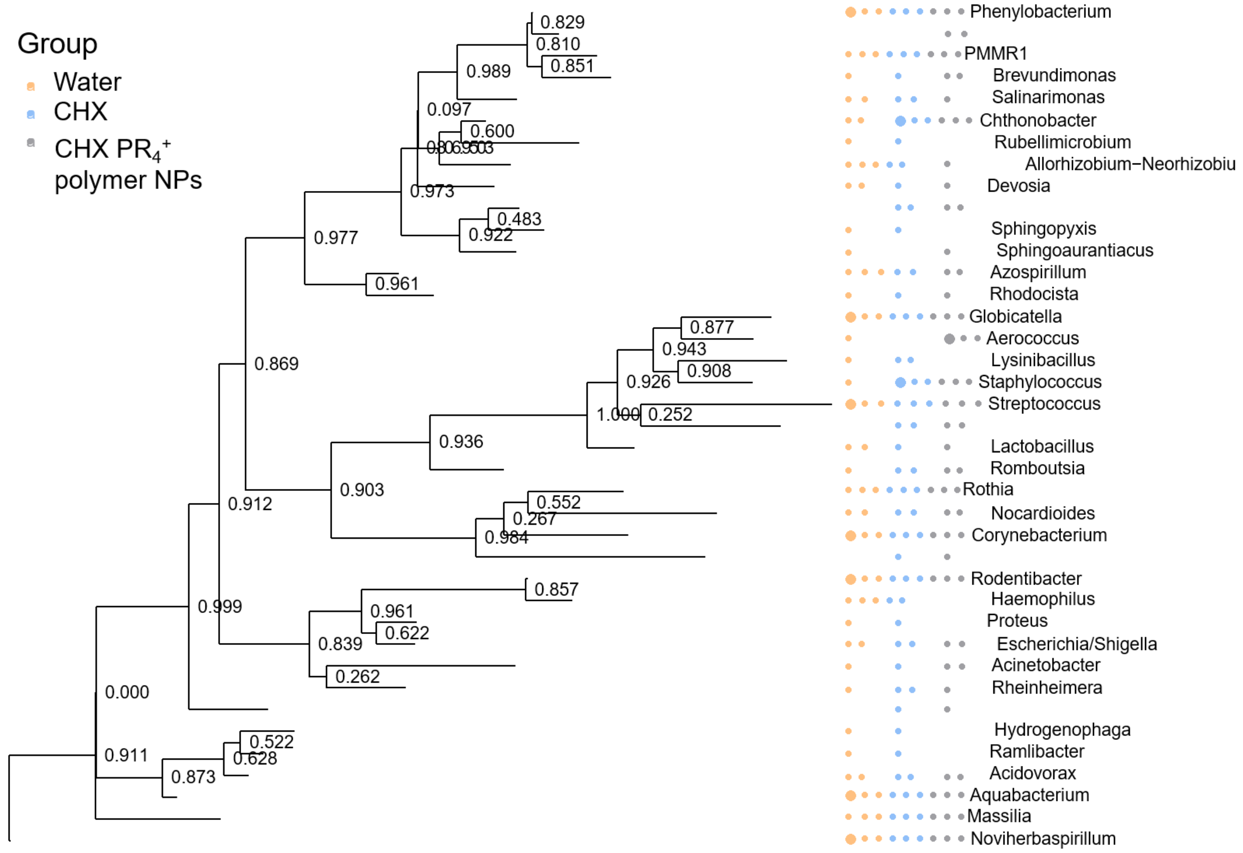


**Figure S28.** Phylogenetic tree based on 20 top highest abundance operational taxonomic unit (OUT).

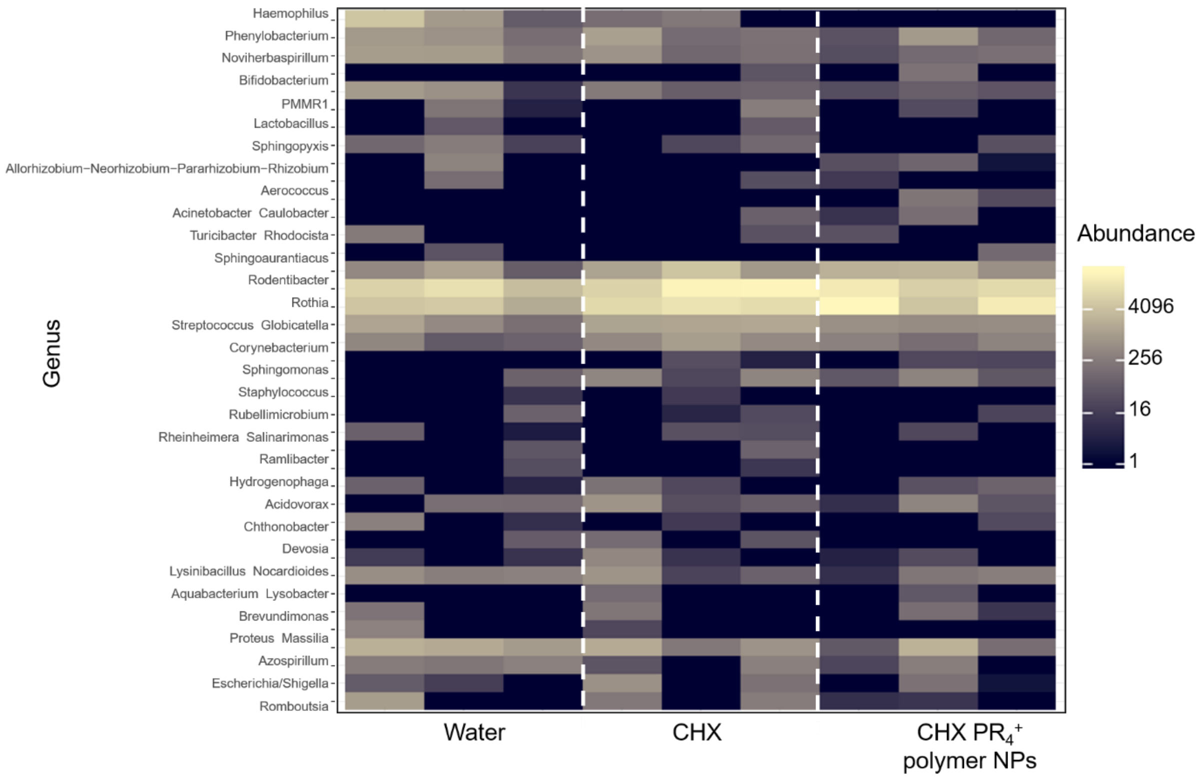


**Figure S29.** Heatmap of overall taxa β-diversity on the glommed samples based on Bray-Curtis distance.

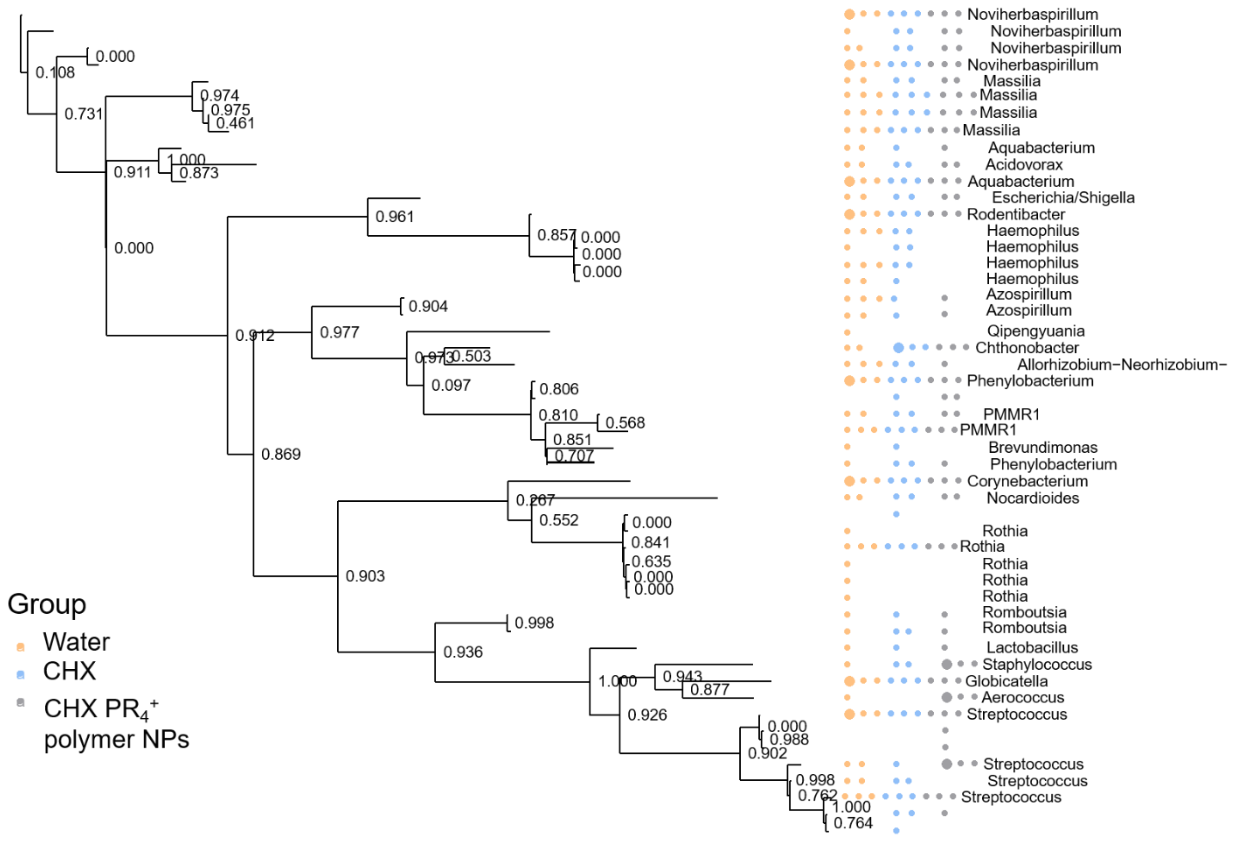


**Figure S30.** Composition tree plot
