# Supplementary material for: Function-adaptive clustered nanoparticles reverse Streptococcus mutans dental biofilm and maintain microbiota balance
Source: Commun Biol. 2021 Jul 15;4:846. doi: 10.1038/s42003-021-02372-y (PMC8282845; doi:10.1038/s42003-021-02372-y)
Supplement: Supplementary file 2 — Description of Supplementary Files [file 42003_2021_2372_MOESM2_ESM.pdf]

Description of Additional Supplementary Files

File name: Supplementary Data 1

Description: Source data underlying plots shown in figures 2-7.

Supplementary Data 12: GO Analysis of Male Cocaine versus Male Saline (All Proteins). GO Terms identified from proteins significantly different in male cocaine versus male saline groups, with relevant statistics.

Supplementary Data 13: REVIGO Analysis of Female Cocaine versus Female Saline (All Proteins). Redundant GO Terms in Supplementary Data 11 were eliminated using REVIGO.

Supplementary Data 14: REVIGO Analysis of Male Cocaine versus Male Saline (All Proteins). Redundant GO Terms in Supplementary Data 12 were eliminated using REVIGO.

Supplementary Data 15: RRHO Ranked List of Male Cocaine versus Male Saline (Log2 Fold Change). Uniprot ID of proteins significantly altered in male cocaine compared to male saline. Rank ordered list was generated and ranked based on Log2 fold change.

Supplementary Data 16: RRHO Ranked List of Female Cocaine versus Female Saline (Log2 Fold Change). Uniprot ID of proteins significantly altered in female cocaine versus female saline. Rank ordered list was generated and ranked based on Log2 fold change.

Supplementary Data 17: RRHO Ranked List Male versus Female Baseline (Log2 Fold Change). Uniprot ID of all proteins identified in the NAc using DIA mass spectrometry. Rank ordered list was generated and sorted based on Log2FC of male proteins compared to female controls at baseline (between saline groups).

Supplementary Data 18: RRHO Ranked List Female versus Male Baseline (Log2 Fold Change). Uniprot ID of all proteins identified in the NAc using DIA mass spectrometry. Rank ordered list was generated and sorted based on Log2FC of female proteins compared to male controls at baseline (between saline groups).
