# Supplementary material for: Function-adaptive clustered nanoparticles reverse Streptococcus mutans dental biofilm and maintain microbiota balance
Source: Commun Biol. 2021 Jul 15;4:846. doi: 10.1038/s42003-021-02372-y (PMC8282845; doi:10.1038/s42003-021-02372-y)
Supplement: Supplementary file 4 — Reporting Summary [file 42003_2021_2372_MOESM4_ESM.pdf]

nature research

Corresponding author(s): Dipanjan Pan

Last updated by author(s): Jun 3, 2021

Reporting Summary

Nature Research wishes to improve the reproducibility of the work that we publish. This form provides structure for consistency and transparency in reporting. For further information on Nature Research policies, see our Editorial Policies and the Editorial Policy Checklist.

Statistics

For all statistical analyses, confirm that the following items are present in the figure legend, table legend, main text, or Methods section.

n/a	Confirmed	
☐	☒	The exact sample size (*n*) for each experimental group/condition, given as a discrete number and unit of measurement
☐	☒	A statement on whether measurements were taken from distinct samples or whether the same sample was measured repeatedly
☒	☐	The statistical test(s) used AND whether they are one- or two-sided<br>*Only common tests should be described solely by name; describe more complex techniques in the Methods section.*
☒	☐	A description of all covariates tested
☒	☐	A description of any assumptions or corrections, such as tests of normality and adjustment for multiple comparisons
☒	☐	A full description of the statistical parameters including central tendency (e.g. means) or other basic estimates (e.g. regression coefficient) AND variation (e.g. standard deviation) or associated estimates of uncertainty (e.g. confidence intervals)
☒	☐	For null hypothesis testing, the test statistic (e.g. *F*, *t*, *r*) with confidence intervals, effect sizes, degrees of freedom and *P* value noted<br>*Give P values as exact values whenever suitable.*
☒	☐	For Bayesian analysis, information on the choice of priors and Markov chain Monte Carlo settings
☐	☒	For hierarchical and complex designs, identification of the appropriate level for tests and full reporting of outcomes
☒	☐	Estimates of effect sizes (e.g. Cohen's *d*, Pearson's *r*), indicating how they were calculated

Our web collection on statistics for biologists contains articles on many of the points above.

Software and code

Policy information about availability of computer code

Data collection	The TEM images were recorded on a Gatan UltraScan 2kx2k CCD. These CCD images were processed and analyzed with ImageJ (http://rsbweb.nih.gov/ij/) version 1.48.
Data analysis	The data were analyzed by GraphPad Prism, Origin, ChemDraw and microsoft powerpoint software. The flow cytometry results were analyzed and plotted using Flowpy.

For manuscripts utilizing custom algorithms or software that are central to the research but not yet described in published literature, software must be made available to editors and reviewers. We strongly encourage code deposition in a community repository (e.g. GitHub). See the Nature Research guidelines for submitting code & software for further information.

Data

Policy information about availability of data

All manuscripts must include a data availability statement. This statement should provide the following information, where applicable:

- Accession codes, unique identifiers, or web links for publicly available datasets
- A list of figures that have associated raw data
- A description of any restrictions on data availability

The authors declare that the data supporting all the findings of this study are available within the article and Supporting Information files. All the other relevant data are available from the author upon reasonable request.

Field-specific reporting

Please select the one below that is the best fit for your research. If you are not sure, read the appropriate sections before making your selection.

☒ Life sciences ☐ Behavioural & social sciences ☐ Ecological, evolutionary & environmental sciences

For a reference copy of the document with all sections, see nature.com/documents/nr-reporting-summary-flat.pdf

Life sciences study design

All studies must disclose on these points even when the disclosure is negative.

Sample size	Female Sprague Dawley rats (5 weeks old) were purchased from Charles River Laboratories (Chicago, IL). All the rats were prescreened for any potential indigenous S. mutans biofilm with a Saliva Check Mutans Kit (GC America Inc, Alsip, IL). SalivaBio infant swab devices (Salimetrics, LLC, Carlsbad, CA, US) were used for sample collection. The rats (n=3) were randomly placed into three treatment groups: 1) water, 2) CHX, and 3) CHX PR4+ polymer NPs.
Data exclusions	No data were excluded from the analyses.
Replication	All attempts for replication of the presented data in this manuscript were successful.
Randomization	The allocation of groups were random.
Blinding	The investigators were blinded during group allocation/ analyses.

Reporting for specific materials, systems and methods

We require information from authors about some types of materials, experimental systems and methods used in many studies. Here, indicate whether each material, system or method listed is relevant to your study. If you are not sure if a list item applies to your research, read the appropriate section before selecting a response.

Materials & experimental systems

n/a	Involved in the study	
☒	☐	Antibodies
☐	☒	Eukaryotic cell lines
☒	☐	Palaeontology and archaeology
☐	☒	Animals and other organisms
☒	☐	Human research participants
☒	☐	Clinical data
☒	☐	Dual use research of concern

Methods

n/a	Involved in the study	
☒	☐	ChIP-seq
☐	☒	Flow cytometry
☒	☐	MRI-based neuroimaging

Eukaryotic cell lines

Policy information about cell lines

Cell line source(s)	ATCC
Authentication	None of the cell lines used were authenticated.
Mycoplasma contamination	The cell line used were not tested for mycoplasma contamination, but cultured with proper aesthetic condition with necessary antimicrobial reagents.
Commonly misidentified lines (See ICLAC register)	The present study does not involve any cancer cell lines those are listed in the register of misidentified cell lines.

Animals and other organisms

Policy information about studies involving animals; ARRIVE guidelines recommended for reporting animal research

Laboratory animals	Female Sprague Dawley rats (5 weeks old) were purchased from Charles River Laboratories (Chicago, IL). All the rats were prescreened for any potential indigenous S. mutans biofilm with a Saliva Check Mutans Kit (GC America Inc, Alsip, IL). SalivaBio infant swab devices (Salimetrics, LLC, Carlsbad, CA, US) were used for sample collection.
Wild animals	The study did not involve wild animals.

Field-collected samples	The study did not involve samples collected from the field.
Ethics oversight	The experimental protocol received ethical approval and was approved by the Institutional Animal Care and Use Committee (IACUC), University of Illinois at Urbana–Champaign, and satisfied all University and National Institutes of Health (NIH) rules for the humane use of laboratory animals. All animal experiments were carried out according to the ethical guidelines outlined by the Illinois Institutional Animal Care and Use Committee at University of Illinois Urbana-Champaign and were approved by the board (protocol number: 17103).

Note that full information on the approval of the study protocol must also be provided in the manuscript.

Flow Cytometry

Plots

Confirm that:

☒ The axis labels state the marker and fluorochrome used (e.g. CD4-FITC).

☒ The axis scales are clearly visible. Include numbers along axes only for bottom left plot of group (a 'group' is an analysis of identical markers).

☒ All plots are contour plots with outliers or pseudocolor plots.

☒ A numerical value for number of cells or percentage (with statistics) is provided.

Methodology

Sample preparation	Before the animals were sacrificed by asphyxiation, they were tested for S. mutans infection with the kit. The major organs, teeth, and gums were fixed in formalin and submitted for histopathological examination. The extracted teeth were immersed in BHI broth, and the bacteria were sonicated off the teeth.
Instrument	Formalin-fixed specimens, including samples of the major organs and gingiva, were dehydrated and embedded in paraffin using a vacuum infiltration tissue processor (Tissue-Tek VIP 6, Sakura Seiki Co, Nagano, Japan). The paraffin-embedded specimens were then sectioned at 3 μm intervals using a microtome, mounted onto glass slides, dewaxed and stained with hematoxylin-eosin (H&E) using an automatic slide stainer (Tissue-Tek Prisma A1D, Sakura Finetek Co, Torrance, CA).
Software	Samples were analyzed using a Guava EasyCyte Plus Flow cytometer. The results were analysed and plotted using Flowpy.
Cell population abundance	To further characterize the cytotoxicity of our delivery platform, cells (NIH3T3, 10000 cells/well) were grown in 96 well plate for 24 hours before treating with different samples.
Gating strategy	For each sample, data from 10000 single cell events were collected for 3 minutes, in triplicates, and forward scatter vs side scatter information were recorded.

☐ Tick this box to confirm that a figure exemplifying the gating strategy is provided in the Supplementary Information.
